# Brain–Behavior Associations for Risk Taking Depend on the Measures Used to Capture Individual Differences

**DOI:** 10.3389/fnbeh.2020.587152

**Published:** 2020-11-17

**Authors:** Loreen Tisdall, Renato Frey, Andreas Horn, Dirk Ostwald, Lilla Horvath, Andreas Pedroni, Jörg Rieskamp, Felix Blankenburg, Ralph Hertwig, Rui Mata

**Affiliations:** ^1^Center for Cognitive and Decision Sciences, Faculty of Psychology, University of Basel, Basel, Switzerland; ^2^Faculty of Psychology, Stanford University, Stanford, CA, United States; ^3^Center for Adaptive Rationality, Max Planck Institute for Human Development, Berlin, Germany; ^4^Movement Disorders and Neuromodulation Section, Charité – University Medicine Berlin, Berlin, Germany; ^5^Computational Cognitive Neuroscience, Free University of Berlin, Berlin, Germany; ^6^Methods of Plasticity Research, University of Zurich, Zurich, Switzerland; ^7^Center for Economic Psychology, University of Basel, Basel, Switzerland; ^8^Neurocomputation and Neuroimaging, Free University Berlin, Berlin, Germany

**Keywords:** risk taking, brain–behavior, fMRI, BART, monetary gambles, nucleus accumbens, individual differences

## Abstract

Maladaptive risk taking can have severe individual and societal consequences; thus, individual differences are prominent targets for intervention and prevention. Although brain activation has been shown to be associated with individual differences in risk taking, the directionality of the reported brain–behavior associations is less clear. Here, we argue that one aspect contributing to the mixed results is the low convergence between risk-taking measures, especially between the behavioral tasks used to elicit neural functional markers. To address this question, we analyzed within-participant neuroimaging data for two widely used risk-taking tasks collected from the imaging subsample of the Basel–Berlin Risk Study (*N* = 116 young human adults). Focusing on core brain regions implicated in risk taking (nucleus accumbens, anterior insula, and anterior cingulate cortex), for the two tasks, we examined group-level activation for risky versus safe choices, as well as associations between local functional markers and various risk-related outcomes, including psychometrically derived risk preference factors. While we observed common group-level activation in the two tasks (notably increased nucleus accumbens activation), individual differences analyses support the idea that the presence and directionality of associations between brain activation and risk taking varies as a function of the risk-taking measures used to capture individual differences. Our results have methodological implications for the use of brain markers for intervention or prevention.

## Introduction

Many events, trajectories, and transitions central to human life are shaped by the extent to which individuals are risk loving or risk averse, particularly in the domains of health, wealth, and criminal activity (Moffitt et al., 2011). Accordingly, individual differences in risk preference (as well as related constructs and constituent factors) present desirable targets for clinical, developmental, and longitudinal research aiming to identify reliable markers for the purpose of intervention and prevention (Conrod et al., 2013). Backed by a recent genome-wide association study that suggested that the genetic basis for domain-general risk taking is predominantly expressed in the brain tissue (Linnér et al., 2019), a prominent approach to understanding and, ultimately, predicting individual differences in risk taking focuses on neural pathways (Sherman et al., 2018).

What do we know about the neural basis of risk taking that may help understand individual differences? Qualitative (Platt and Huettel, 2008; Bjork and Pardini, 2014; Knutson and Huettel, 2015; Samanez-Larkin and Knutson, 2015) and quantitative reviews (Mohr et al., 2010; Wu et al., 2012; Bartra et al., 2013) of studies that have used functional magnetic resonance imaging (fMRI) to examine brain function in response to behavioral measures of risk point towards several brain regions of interest, converging in particular on increased activation in the nucleus accumbens (NAcc), (anterior) insula (AIns), and anterior cingulate cortex (ACC) as key functional correlates of risk taking. Furthermore, a large-scale term-based meta-analysis of fMRI studies (Yarkoni et al., 2011) also points toward increased activation in the NAcc, AIns, and ACC as *consistently* and *preferentially* associated with the term “risk taking” (accessed September 5, 2020).^1^ Regarding the underlying mechanisms, these regions have been suggested to constitute the core elements of a neural *risk matrix* (Knutson and Huettel, 2015), the differential activation in which is thought to facilitate the promotion (NAcc in ventral striatum), inhibition (AIns), and control (ACC) of risky choice.

Regional brain activations in response to risky versus safe decisions alone do not necessarily reflect useful or reliable predictors for the outcomes of interest (Poldrack et al., 2018), however, and instead a combination of within-participant designs and individual differences analyses is required (Foulkes and Blakemore, 2018). Empirical research on neural functional markers for individual differences in risk taking and related constructs abound (Paulus et al., 2003; Bjork and Pardini, 2014; Helfinstein et al., 2014; Braams et al., 2015; Qu et al., 2015; Büchel et al., 2017; Blankenstein et al., 2018; Casey et al., 2018; MacNiven et al., 2018; Krönke et al., 2020); synthesis of the available evidence indicates heterogeneous findings for both the presence and directionality of associations between neural function and risk-related outcomes (Sherman et al., 2018). For example, risk-related brain activation in the NAcc was found to be positively and negatively associated with self-reported risk taking (Congdon et al., 2013), and despite contributing to the successful classification of risky versus safe choices (Helfinstein et al., 2014), risk-related AIns activation was both positively and negatively associated with risky choice (Paulus et al., 2003; Telzer et al., 2015).

How can we account for the mixed findings relating brain activation with (real-life) risk taking? One crucial aspect lies in the circumstance that much of our current understanding of the association between neural function and risk-related outcomes is synthesized across a wide range of risk-taking measures used to both elicit brain activation and define outcome measures of interest (Sherman et al., 2018). Indeed, numerous behavioral measures and self-report inventories believed to capture individual differences are available (Appelt et al., 2011; Dohmen et al., 2011), yet within-participant (psychometric) designs suggest that different measures result in weakly or uncorrelated estimates of individuals’ risk preferences as a likely result of their idiosyncratic recruitment of cognitive and affective processes (Frey et al., 2017; Pedroni et al., 2017; van den Bos and Hertwig, 2017; Pabon et al., 2019). For example, a common distinction between behavioral measures rests on whether decision-relevant information (e.g., range and mean of probabilities, gains, and losses) is mostly unavailable and thus ambiguous, available in the form of fully described properties, or in principle available but must be learned through repeated experience. Although such ambiguity, description, and experience-based risk-taking measures, respectively, share central characteristics (e.g., the presence of uncertainty, integration of available information into a subjective value signal), they differ in the involvement of further requisite or incidental processes, including learning, attention, affect, and memory (Figner et al., 2009; Hertwig and Erev, 2009; Rosenbaum et al., 2018). Perhaps unsurprisingly, the heterogeneous trajectories found for risk taking across the life span are, in parts, reflective of the way in which risk is encountered and how the relative cognitive (and affective) demands of the measures used to operationalize risk interact with age (Mata et al., 2011; Tymula et al., 2012, 2013; Li et al., 2013; Frey et al., 2015a; Mamerow et al., 2016; Rosenbaum et al., 2018).

In the context of fMRI studies, recent reviews have highlighted the issue of measurement, that is, how brain markers are elicited and how risk-related outcomes of interest are assessed, as a central problem in need of clarification (Sherman et al., 2018; Bjork, 2020). Interestingly, even though numerous behavioral measures are used to elicit risk-related neural functional markers (Mohr et al., 2010; Wu et al., 2012; Bartra et al., 2013; Knutson and Huettel, 2015), only a few fMRI studies have empirically compared different measures in within-participant designs with regard to group-level brain activation and individual differences analyses (FitzGerald et al., 2010; Congdon et al., 2013; Pletzer and Ortner, 2016). For example, a conjunction analysis of group-level activations associated with experience-based risk taking in the Balloon Analog Risk Task (BART) and description-based risk taking in the Game of Dice Task (Pletzer and Ortner, 2016) revealed joint activation increases in bilateral striatal regions and insula, yet also a stronger “Risky > Safe” contrast in an extensive frontoparietal network for the Game of Dice Task relative to the BART, which the authors suggest reflects more reflective risk taking in the former compared with the latter task. In addition to relatively small sample sizes, however, a common shortcoming of previous fMRI studies that have used a two-task, within-participant design is the use of single indices of the risk-related outcomes that are examined for their association with neural function.

Here, we investigated whether brain activation in the NAcc, AIns, and ACC elicited within participants from two popular behavioral risk-taking measures in a large sample of young adults is differentially associated with various indicators of (real-life) risk taking. In particular, we used fMRI versions of the BART (Lejuez et al., 2002; Schonberg et al., 2012) and monetary gambles (Tom et al., 2007) to elicit risk-taking related neural functional markers. Importantly, both tasks feature similar concepts such as loss, reward, and risk; yet, whereas these parameters are explicitly described for monetary gambles, some must be explored and learned through experience in the BART (Wallsten et al., 2005; Pleskac, 2008). In using these two fMRI tasks, our aim is not to neurally dissociate experienced from described risk, for this question has already been addressed using well-suited and meticulously controlled experimental designs (FitzGerald et al., 2010). Instead, the aim of our two-task approach is to examine, at the level of brain–behavior associations for two ubiquitous fMRI risk-taking tasks, what has already been shown psychometrically (Frey et al., 2017, 2020; Pedroni et al., 2017), namely that risk-taking measures should not be used interchangeably.

Guiding our analyses were the following research questions (RQ). RQ1: Do the two fMRI tasks elicit common group-level activation for risky versus safe decisions in *risk matrix* regions? Previous literature (Pletzer and Ortner, 2016) examining similar tasks suggests that joint activation increases could be especially evident in the striatum and insular cortex. RQ2: To what extent are functional markers positively correlated across the two fMRI measures at the level of the individual? Owing to the often-neglected lack of a match between group- and individual-level effects (Blanco et al., 2011; Bornstein et al., 2017; Fisher et al., 2018), any observed convergence of group-level neural function does not necessarily indicate individual-level consistency (Fliessbach et al., 2010; Elliott et al., 2020; Korucuoglu et al., 2020; Li et al., 2020). RQ3: To what extent do brain–behavior associations change as a function of how risk-related neural activation is elicited and risk-related outcomes are assessed? Considering the ongoing debate on the nature, structure, and dimensionality of risk preference (Frey et al., 2017; Mata et al., 2018; Eisenberg et al., 2019; Hertwig et al., 2019; Pabon et al., 2019), how risk-related outcomes are assessed poses a challenge for associations with brain function (Sherman et al., 2018). To address this issue, first, we examined within-task brain–behavior associations because this provides insight into the neural mechanisms mapping onto, and ideally giving rise to, behavior on a given task. Second, we examined the extent to which neural markers from one fMRI task were associated with performance on the other because such out-of-task associations could be suggestive of more general mechanisms being captured. Third, we investigated associations between neural function and latent measures of risk preference obtained from psychometric modeling of a large battery of risk-taking measures collected out of session. Latent variables promise to reflect more error-free and thus more reliable measures of risk preference, and by correlating these with neural indices, we can test to what extent the three *risk matrix regions* are differentially associated with more refined indices of risk preference.

The functional significance and dissociation of activation in the NAcc, AIns, and ACC for risky choice, as postulated by the *risk matrix* framework (Knutson and Huettel, 2015), leads to the hypotheses that risk-related outcomes should be positively associated with risk-taking-related NAcc activation and negatively associated with risk-related activation in the AIns and ACC. However, to the extent that the two fMRI tasks vary on task-specific demands that may moderate brain–behavior associations (Schonberg et al., 2011; Congdon et al., 2013; Sherman et al., 2018; Bjork, 2020), we explore whether and how the resultant associations between brain function and risk-related outcomes differ from the associations expected as per the *risk matrix* framework. Intuitively, the two fMRI tasks used here differ with regard to how risk is encountered, which in turn is likely to affect attentional demands (BART > monetary gambles), affective responses (BART > monetary gambles), and feedback-based integrative processes (BART > monetary gambles), among others. However, a formal comparison of our task implementations (e.g., facilitated by a validated taxonomy of risk-taking measures) is still outstanding; in its absence, our findings will help generate focused hypotheses about the impact of task-specific aspects on brain–behavior associations.

## Materials and Methods

### Experimental Design

#### Participant Recruitment

Participants in this fMRI study were recruited from an existing pool of individuals who had participated in the Basel–Berlin Risk Study (BBRS). The BBRS is a large-scale (*n* = 1,507) study assessing individual differences, psychometric structure, and biological underpinnings of risk preference (Dutilh et al., 2017; Frey et al., 2017; Pedroni et al., 2017). Participants in the BBRS completed an extensive battery of risk-taking measures (including self-report and behavioral measures), as well as other individual differences measures, including cognitive capacity, personality, affect, and genetics (an overview of all subsamples, measures, and further details on the BBRS is reported on https://osf.io/rce7g). The BBRS was run in Basel (Switzerland) and Berlin (Germany), but we recruited only individuals from the Berlin site for the imaging study due to the location of the neuroimaging facilities available. Exclusion criteria for participation in the neuroimaging study were safety-limiting permanent implants, a history of neurological or psychiatric conditions, usage of psychoactive medication or substances, and receiving psychiatric treatment.

Reflective of oversampling to achieve an effective sample size of *N*∼100 (Yarkoni, 2009) in the event of participant exclusions (e.g., due to excessive head motion in the scanner, image artifacts), we recruited a total of 133 participants. Two participants aborted the session before any functional sequences were collected and were removed from all subsequent analyses. A further five participants were excluded due to excessive head motion inside the scanner (see image preprocessing section for movement parameter thresholds), one participant due to incidental anatomical findings, four participants due to incomplete data (e.g., only one of the two risk-taking measures was completed inside the scanner), and five participants for non-compliance with the scanner protocol (e.g., falling asleep, reports of having mixed up button box responses). The final sample included in all analyses comprised 116 participants (62 females; mean age at scan = 25.34 years; *SD* = 2.64 years; range = 20.4–30.1 years).

#### Experimental Procedure and Compensation

Participants who had completed the BBRS laboratory session were contacted via phone and informed about the MRI follow-up study. Interested individuals were screened via telephone for any contraindications regarding MRI safety and invited to participate if no exclusion criteria applied. At the time of the imaging session, all individuals were screened again for contraindicators, followed by a 2-min training run for each of the two fMRI tasks (BART and monetary gambles) before entering the scanner. The scanner protocol took 75 min and included a high-resolution structural scan, two functional sequences for the BART, two functional sequences for monetary gambles, a resting-state sequence, and a diffusion-weighted imaging sequence. For the current study, only the high-resolution structural scan and the functional sequences were utilized, with the structural scan only serving normalization purposes during preprocessing of functional imaging data. The resting-state and diffusion-weighted sequences were not part of the current analysis and are not discussed further. The fMRI tasks were presented using E-Prime 2.0 software (Psychology Software Tools, Pittsburgh, PA), and responses inside the scanner were collected via a COVILEX response box system (series 1.X, Magdeburg, Germany) using the right-hand index and middle finger. After the MRI session, individuals reported demographic data and completed additional measures (see section “Experimental Measures”).

At the end of the session, participants received a fixed fee of 25 Euro for their participation. In addition, participants could increase their earnings based on performance in the two fMRI tasks. For the BART, participants received 0.05 Euro for each successful pump on a balloon that was cashed out, i.e., did not explode. For monetary gambles, one trial was drawn at random and, if the participant had accepted the trial, was played out. The resulting loss or gain was combined with money made in the BART. Trials that were drawn but which the participant had rejected resulted in a 0-Euro outcome. Participants were told about the incentive structure at the start of the MRI session and received cash earnings at the end of the session (average actual payment = 41.50 Euro, *SD* = 14.50 Euro).

#### Experimental Measures

**FIGURE 1:**
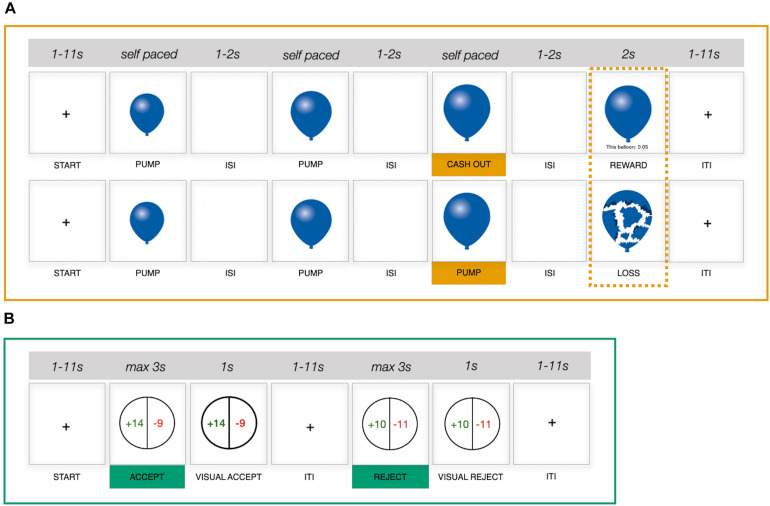
Schematic representation of two functional magnetic resonance imaging (fMRI) risk-taking measures. **(A)** Balloon Analog Risk Task (BART), *upper row*, example cash-out trial; *lower row*, example explosion trial. **(B)** Monetary gambles. ISI, interstimulus interval; ITI, intertrial interval; s, seconds.

The fMRI session involved incentivized, performance-compatible versions of two prototypical measures of experience- and description-based risk taking (Figure 1), which have been used in neuroimaging research and investigated extensively with regard to group-level neural activation profiles (Tom et al., 2007; Rao et al., 2008; Schonberg et al., 2012; Canessa et al., 2013; Pletzer and Ortner, 2016; Botvinik-Nezer et al., 2020) and individual differences (Tom et al., 2007; Canessa et al., 2013; Peper et al., 2013; Helfinstein et al., 2014; Braams et al., 2015; Pletzer and Ortner, 2016).

##### Balloon Analog Risk Task

The BART (Lejuez et al., 2002) involves a series of virtual balloons, which individuals are tasked with pumping up in the absence of knowledge about when the balloon will burst. Successful pumps (i.e., pumps that do not lead to a balloon explosion) earn the participant money, but an explosion leads to the loss of the money accumulated on the current trial. Individuals thus make repeated decisions about whether to (1) continue pumping up a balloon (i.e., risky decision), with the prospect of accumulating more money, or (2) stop pumping and cash out any accumulated earnings on a given trial (i.e., safe decision), yet foregoing any further earnings on that trial. Importantly, as individuals move from trial to trial and experience the outcome of their decisions (e.g., a balloon explosion), they can build a mental representation of explosion distributions for a given balloon type over time. The BART version implemented in the current study featured two risky balloon types and a control balloon. The maximum capacity for the two risky balloons was set to be 12 and 20 pumps, respectively; that is, on average, balloons with a capacity of 12 pumps burst earlier than balloons with a capacity of 20. Risky balloons were represented in blue and red to discriminate between balloon types based on capacity, with capacity–color assignment being randomized between participants but stable across the two runs. Control balloons were presented in gray, had a maximum capacity of 16, and were added to control for neural processes that required no decision making (e.g., motor or visual). Participants merely inflated control balloons until they disappeared from the screen. On any given trial, balloon capacity was determined via a random draw from a uniform distribution of values between one and the maximum capacity for the presented balloon type. Participants completed two runs of the BART, with a short break in between. Each run was programmed to continue for 10 min, after which the final balloon was presented. Given that decisions are made sequentially and may become more difficult as the number of successful pumps in a trial increases, we did not impose a time limit on the decision phase of a given trial, resulting in the number of balloons played varying between individuals (Supplementary Table 1). Intervals between trials and between successive stimuli within trials were randomized (mean intertrial interval = 4.39 s, range = 1–11 s; mean interstimulus interval = 1.5 s, range = 1–2 s).

To disentangle different cognitive processes underlying the observed behavior in the BART, including gain and loss sensitivity, response consistency, risk preference, or learning (Wallsten et al., 2005; van Ravenzwaaij et al., 2011), we fitted two standard computational models: a target model that assumes a fixed strategy is being used (Pleskac, 2008; Frey et al., 2015b) and a Bayesian sequential risk-taking model that allows for dynamic updating processes (Pleskac, 2008). In line with past research (van Ravenzwaaij et al., 2011), however, the estimation of the model parameters turned out to be unreliable, and we thus do not report the modeling attempt here (a possible reason for the unreliable model parameters may be the lack of strong learning effects). Consequently, we relied on the average number of pumps as a simpler and generic index of risk preference in all subsequent analyses (apart from the mixed-effects trial-by-trial modeling of choice behavior).

##### Monetary gambles

**FIGURE 2:**
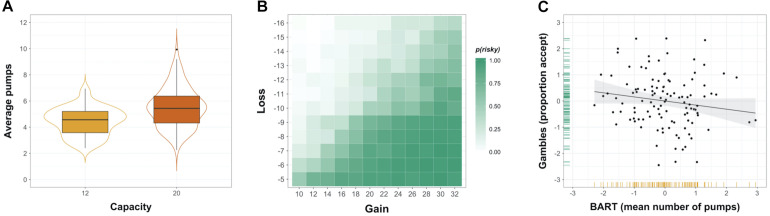
Risk taking in two common behavioral measures. **(A)** Distribution of individuals’ average number of pumps in the Balloon Analog Risk Task (BART), shown separately for balloons with capacity 12 and 20. **(B)** Payoff matrix overlaid with heatmap showing the observed probability of gamble acceptance in monetary gambles. **(C)** Association between risk taking in the BART and monetary gambles (plotted are the standardized residuals after regressing out effects of age and gender), with variable distributions shown in the margins.

We adopted a monetary gambles paradigm with mixed outcomes as an example of a description-based risk-taking measure (i.e., both gains and losses were possible) (Tom et al., 2007; Barkley-Levenson et al., 2013; Canessa et al., 2013; Sokol-Hessner et al., 2013). In the current study, participants made a total of 144 decisions between a sure zero outcome and a 50/50 gamble without feedback (i.e., gamble outcomes were not presented). Individual gambles were constructed to populate an asymmetric 12 × 12 payoff matrix (Figure 2B) with gains between 10 and 32 (increments of 2) and losses between 5 and 16 (increments of 1). Each gamble was presented once, with the order of gamble presentation randomized between participants. On a given trial, once the gamble was presented, participants had 3 s to accept or reject the gamble via respective button presses. Although in previous studies participants gave responses indicating the strength of their decision (Tom et al., 2007; Canessa et al., 2013), we collected binary responses (accept/reject) only. Considering that previously reported analyses were commonly conducted on collapsed (binary) responses (Tom et al., 2007; Canessa et al., 2013), we did not expect a substantial benefit from adopting more fine-grained response options. Participants completed two runs with a short pause in between, each run featuring 72 gambles. Jitters were introduced between trials (mean intertrial interval = 4.32 s, range = 1–11 s).

A simple model that captures sensitivity to gains versus losses has been used to capture decision making for monetary gambles (Tom et al., 2007; Barkley-Levenson et al., 2013; Canessa et al., 2013). However, the critical parameter of this model, loss aversion, was negatively correlated with the proportion of accepted gambles (*r* = *-*0.90, *p* < 0.001). Consequently, we relied on the proportion of accepted gambles as a simpler and generic index of risk preference in all subsequent analyses (apart from the mixed-effects trial-by-trial modeling of choice behavior).

##### Psychometric risk preference factors

Behavioral measures often suffer from poor test–retest reliability (Frey et al., 2017; Enkavi et al., 2018) and (accordingly) low convergence (Frey et al., 2017; Pedroni et al., 2017); thus, selecting single measures as indicators of risk preference may constrain the utility of examining associations with neural function (Sherman et al., 2018). To address this issue, we used psychometric factors that were extracted across 39 widely used risk-taking measures collected from the full BBRS sample via implementation of a bifactor model; see Frey et al. (2017) for a comprehensive list of measures and details on latent variable modeling analyses. The bifactor model gave rise to a general risk preference factor, *R* (akin to the general factor of intelligence), that captured 61% of the explained variance across risk-taking measures and seven specific orthogonal factors that captured additional domain- or situation-specific variance. The domain-specific factors were suggested to represent attitudes and behaviors associated with health risk taking (F1), financial risk taking (F2), recreational risk taking (F3), impulsivity (F4), traffic risk taking (F5), occupational risk taking (F6), and choices among (monetary) lotteries (F7). For a subset of BBRS participants (n = 109), test–retest reliability was higher for the psychometric factors than for behavioral measures; for example, the general risk preference factor *R* was observed to have a 6-months retest reliability of 0.85, whereas many of the behavioral measures tested yielded retest reliabilities below 0.5.

Due to a temporal overlap between the end of behavioral data collection in the laboratory and the start of the MRI component, individuals were contacted with varying delays after having completed the BBRS laboratory component. As a result, the MRI sample was heterogeneous with regard to the delay between the laboratory and MRI session (mean delay = 196 days, *SD* = 121 days, range = 1–453 days). Furthermore, the laboratory session of the BBRS took place prior to the MRI session; hence, we refrain from using the term “prediction” (in the strictest sense we could use the term “postdiction”) and instead refer to our analyses as out-of-session associations.

##### Further measures

Outside the scanner, we collected self-reported demographic data (date of birth, gender, marital status, educational attainment, native language, and current occupation). Of note, only gender and age at the MRI session (calculated from date of birth) were included as covariates in the current analyses; all other demographic measures were merely collected to describe the sample and ascertain the external validity of our findings with respect to sample characteristics. As part of an independent project, we assessed individuals’ height and weight, collected data from a verbal fluency task, as well as various self-report measures of impulsivity and eating-related behaviors and attitudes; given that these measures were not part of the current analyses, we do not elaborate on these measures here.

#### MRI Data Acquisition

Neuroimaging data were collected at the Magnetic Resonance Imaging Laboratory at the Max Planck Institute for Human Development (Berlin, Germany) on a 3T Siemens MRI system with 12-channel head coil. Participants were scanned with a magnetization-prepared rapid gradient echo sequence (repetition time = 2,500 ms, echo time = 4.77 ms, inversion time = 1,100 ms, flip angle = 7°, field of view = 256 mm × 256 mm, 192 slices, voxel size = 1 mm × 1 mm × 1 mm). In each of the four functional runs, up to 320 functional T2^∗^-weighted blood–oxygen-level-dependent imaging (BOLD) echo-planar images were acquired for every person (repetition time = 2,010 ms, echo time = 30 ms, flip angle = 78°, field of view = 192 mm × 192 mm, voxel size = 3 mm × 3 mm × 3 mm, 33 transversal slices/volume with 15% distance factor).

### Statistical Analysis

All behavioral analyses were performed in R (R Project for Statistical Computing^2^; RRID:SCR_001905), using the packages lme4 (lme4: linear mixed-effects models using Eigen and S4; R package v 1.1–8)^3^ and lmerTest (lmerTest: tests in linear mixed effects models; R package v 2.0–25).^4^ We used the functions lmer and glmer for the mixed-effects models of continuous and binary outcome variables, respectively. To obtain *p*-values for the fixed-effects test statistics in lmerTest, the calculation of the denominator degrees of freedom adopts Satterthwaite’s approximation (cf. SAS proc mixed theory).

#### Behavioral Analysis

To examine whether risk taking elicited in the BART and monetary gambles mirrored behavioral patterns observed in the literature (Tom et al., 2007; Schonberg et al., 2012; Mamerow et al., 2016), we assessed group-level behavior and performed analyses ascertaining and accounting for the effect of individual as well as contextual variables. The outcome variable typically used in the BART to reflect individuals’ risk preference is the average number of pumps administered on cash-out trials only (Lejuez et al., 2002; Wallsten et al., 2005; Yu et al., 2016), also referred to as the adjusted average number of pumps. In line with previous research (Mamerow et al., 2016; Frey et al., 2017), the adjusted average number of pumps was highly correlated with the average number of pumps across all balloons (*r* = 0.97, *p* < 0.001) as well as for the two balloon capacities separately (*r_12_* = 0.92, *p* < 0.001, *r_20_* = 0.95, *p* < 0.001). Given these results, we used the average number of pumps across all balloons as outcome variable in the BART because it allowed us to retain a maximum number of trials for analysis while working with congruent trial numbers in both neural and behavioral analyses. We dropped the gray control balloons from all behavioral analyses of the BART, as these balloons did not require a choice to be made and are thus uninformative for determining effects on (risky) choice.

To assess whether the experimental manipulation of balloon capacity elicited different risk-taking behavior, we compared the mean number of pumps between balloons with a capacity of 12 and 20 by means of a two-sample paired *t*-test with significance level of *p* < 0.05. In order to estimate the effect of individual and contextual factors on trial-by-trial risky choice, we applied mixed-effects regression analysis. We regressed the number of pumps on reward balloons (for a given trial) on trial-specific fixed effects of balloon capacity as a proxy for the level of risk (0 = 12, 1 = 20), having experienced an explosion on the previous trial (0 = no, 1 = yes), and trial number (continuous). We also included individual differences in age (continuous) and gender (0 = male, 1 = female) in the regression model. To account for individual differences to specific contextual factors, we allowed for random slopes for balloon capacity, trial number, and explosion on the previous trial. Prior to fitting the regression model, all continuous predictor variables were standardized (i.e., centered and scaled by the standard deviation) and categorical variables were dummy coded. The specification of the regression model and selection of predictor variables followed previously reported trial-level effects (Wallsten et al., 2005; Mamerow et al., 2016).

For monetary gambles, we computed the proportion of accepted gambles out of all gambles for which a response was provided as an index of risk taking. In a first step, we used this index to compute the probability of an “Accept” decision for a given option for the entire sample. In a second step, we specified a logistic mixed-effects model, in which the decision to accept or reject (0 = Reject, 1 = Accept) a particular lottery in a given trial was regressed on fixed trial-specific effects of the magnitude of the gain, the absolute magnitude of the loss, the interaction between gain and loss, and individual effects of age (continuous) and gender (0 = male, 1 = female). In the regression model, we allowed random slopes for gain and loss magnitude. All continuous predictor variables were standardized prior to fitting the model, and categorical variables were dummy coded. Model specification was informed by previously reported trial-level effects (Tom et al., 2007). Given differential effects of age and gender on trial-by-trial decision making, we conducted all subsequent analyses on standardized and residualized (i.e., regressing out effects of age and gender) behavioral indices. As a final step in the behavioral analysis, we computed the correlation between mean number of pumps in the BART and proportion of accept decisions in monetary gambles to ascertain the convergence of experience- and description-based risk taking (Figure 2C).

#### Neuroimaging Analysis

Image preprocessing and analyses were carried out using standard procedures implemented in SPM8.^5^ See Supplementary Section 1.1 for details.

##### fMRI model specification

At the level of the individual, we concatenated the two runs for each of the two risk-taking measures and specified one general linear model for the BART and one for monetary gambles. Our aim was to target and compare the neural representation of risky versus safe decisions in both measures, which we operationalized as decisions to administer a pump on reward balloons in the BART and decisions to accept a lottery in monetary gambles. For the main contrast of interest—risky versus safe decisions—we followed standard approaches and contrasted pumping on reward balloons with pumping on control balloons (“Pumps reward versus Pumps control”) in the BART (Rao et al., 2008; Schonberg et al., 2012; Yu et al., 2016) and contrasted decisions to accept a lottery with decisions to reject (“Accept versus Reject”) in monetary gambles (Barkley-Levenson et al., 2013). We considered rejection of a gamble the safe option because by rejecting a gamble, participants implicitly opted for a sure outcome of zero.

To facilitate contrast analyses that tackle RQ1, the individual-level BART GLM included eight regressors (Supplementary Figure 1A for an example individual-level design matrix). We included onset vectors for control, low-capacity, and high-capacity balloons and one onset vector for cash-out events and one onset vector for explosion events (in that order). For each balloon onset vector, we also included a parametric regressor number to facilitate supplementary analyses to better understand the impact of contrast choice on BART brain–behavior associations (Supplementary Section 1.3). Cash-out and explosion events were included to account for additional variance and thus better isolate the main effects of interest by removing neural responses to cash out and explosions from baseline activity. Six motion parameters estimated during the realignment procedure were included as regressors of no interest. The individual-level GLM for monetary gambles included the following regressors: onset vector for all Accept decisions, onset vector for all Reject decisions, and six motion parameters estimated during the realignment procedure (Supplementary Figure 1B for example individual-level design matrix). Multicollinearity between choice and gain/loss magnitudes (and as a consequence, also the expected value of a gamble) reduced the scope of the design matrix. However, the simplicity of the paradigm allowed for this comparatively straightforward design matrix with only two regressors of interest, nevertheless yielding clean (event-unrelated) baseline activity. All analyses involved modeling the time from trial onset (i.e., display of stimulus) until choice (i.e., pump reward/pump control in the BART and accept/reject for monetary gambles).

At the level of the group, we specified two one-sample *t*-tests, one for every measure, in which we tested whether the group-level signal in response to risky versus safe decisions (as operationalized at the level of the individual design matrix) was significantly different from zero, controlling for the effects of age and gender (see Supplementary Section 1.2 for contrast weights). Whole-brain, voxel-wise analyses of group-level activation in the two measures was corrected using family-wise error (FWE) correction at voxel (i.e., peak) level, with a significance level of *p* < 0.05.

##### Regions of interest

**FIGURE 3:**
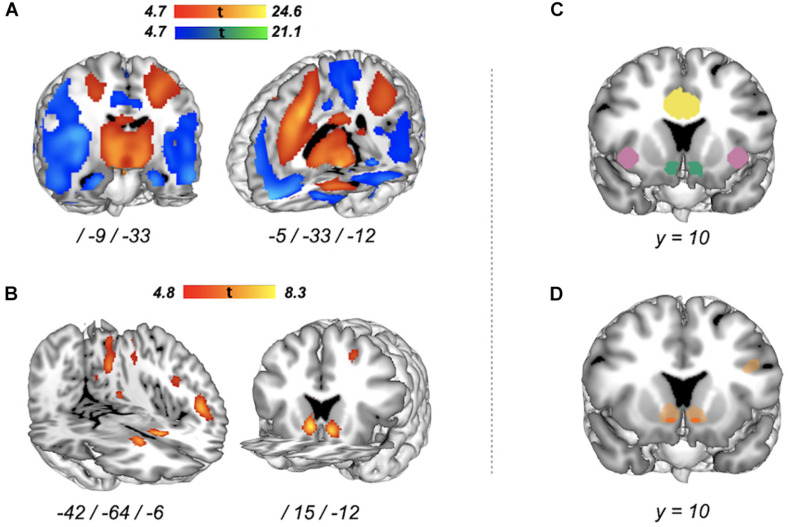
Whole-brain voxel-wise group-level activation for risky versus safe decisions in two functional MRI (fMRI) tasks and their intersection. **(A)** Balloon Analog Risk Task (BART) brain activation [peak-level family-wise error (FWE), *p* < 0.05] for “Pumps reward versus Pumps control”. Red–yellow areas represent increased activation, blue–green areas represent decreased activation. **(B)** Monetary gambles brain activation (peak-level FWE, *p* < 0.05) for “Accept versus Reject”. Red areas represent increased activation (no significant reverse effect). **(C)** Regions of interest (ROIs). Colors correspond to the following ROIs: blueish green for NAcc, reddish purple for AIns, and yellow for ACC. **(D)** Intersection of increased activation associated with risky versus safe decisions in the BART (Pumps reward > Pumps control) and monetary gambles (Accept > Reject). Given our focus on *risk matrix* regions, in dark orange, we show intersecting increased activation for regions of interest only (small volume correction, peak-level FWE, *p* < 0.05). Shown in light orange are neural regions with intersecting increased activation at the level of the whole brain (peak-level FWE correction, *p* < 0.05).

**FIGURE 4:**
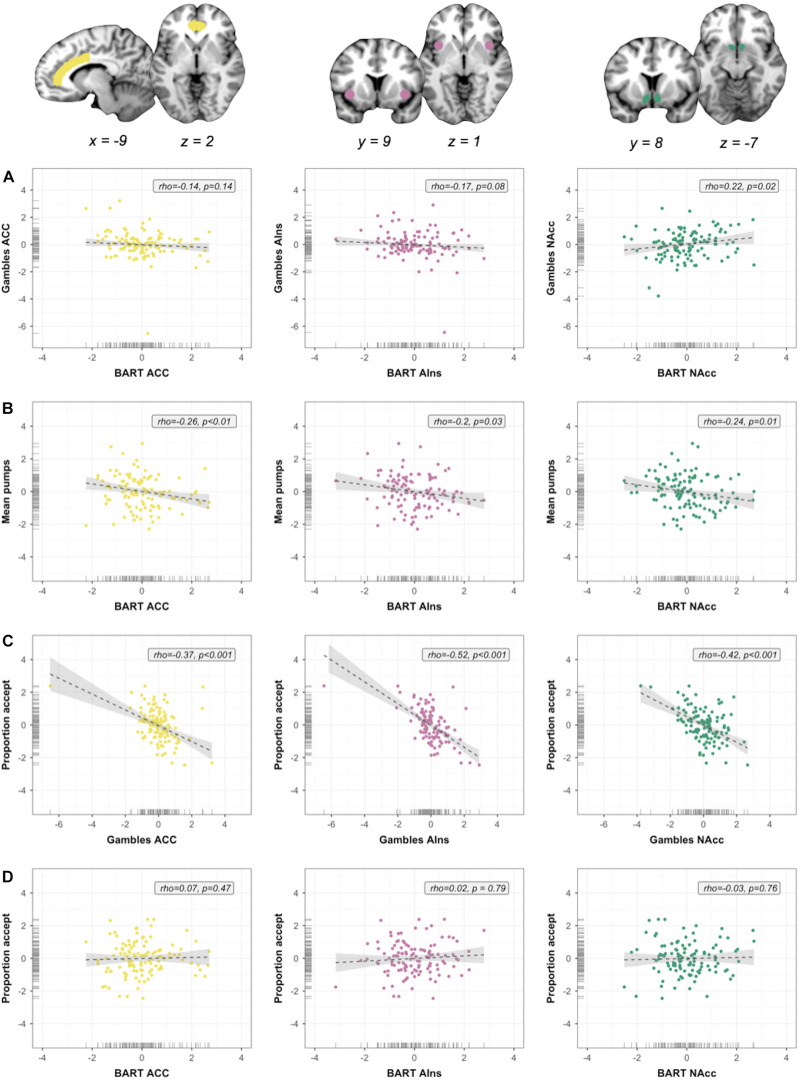
Within-session individual differences. Scatterplots show associations between neural indices, as well as between neural and behavioral indices. Columns represent neural signal extracted from three ROIs [anterior cingulate cortex (ACC), anterior insula (AIns), and nucleus accumbens (NAcc), in that order]; rows represent analyses. **(A)** Associations between regional neural signals across measures (brain–brain). **(B)** Within-measure brain–outcome associations for Balloon Analog Risk Task (BART). **(C)** Within-measure brain–outcome associations for monetary gambles. **(D)** Out-of-measure brain–outcome associations. *Note:* All variables were regressed on age and gender and standardized prior to plotting and analysis. Regression slopes were estimated using robust regression analyses.

To avoid inflated brain–behavior associations that would result from selecting regions of interest (ROIs) based on within-experiment contrast activations (Poldrack and Mumford, 2009; Vul et al., 2009), we selected ROIs *a priori* on the basis of a comprehensive literature review and term-based meta-analysis (see section “Introduction”). We used the Hammersmith atlas nr30r83^6^ to structurally localize the ROIs for the NAcc (volume across left and right hemisphere = 114 voxels) and ACC (volume across left and right hemisphere = 2,660 voxels). In the absence of a validated structural parcelation of the insular cortex, but given our specific focus on the anterior insula, we created an anterior insula ROI (volume across left and right hemisphere = 358 voxels) based on a published meta-analysis of resting-state-based functional connectivity studies (Chang et al., 2013); see Supplementary Section 1.4 for details about the construction of the AIns ROI. The resulting ROIs for NAcc, AIns, and ACC are depicted in Figure 3C and at the top of Figure 4.

For each ROI, we extracted the mean of the regression slopes from each individual’s first-level BART contrast “Pumps reward > Pumps control” and monetary gambles contrast “Accept > Reject”; mean signal for a particular ROI was operationalized as the mean of all regression slopes extracted from all voxels contained in a given ROI. As we had no hypotheses about hemispheric differences in the NAcc, AIns, and ACC, we computed a mean signal across the two hemispheres for each ROI, yielding six summary indices for ROI activation for each participant (i.e., three per fMRI task). High positive correlations between signal extracted from the left and right hemisphere for NAcc (*r*_*BART*_ = 0.95, *r*_*Gambles*_ = 0.88), AIns (*r*_*BART*_ = 0.72, *r*_*Gambles*_ = 0.90), and ACC (*r*_*BART*_ = 0.94, *r*_*Gambles*_ = 0.97) validated the use of a mean signal.

##### Conjunction analysis of group-level activation between tasks

To examine the intersection of neural markers between the two fMRI tasks at group level (RQ1), we conducted a voxel-wise conjunction analysis of activation for risky versus safe decisions in the BART (pumping on reward relative to control balloons) and monetary gambles (accepting relative to rejecting an offer). Specifically, we performed a conjunction analysis over two orthogonal contrasts that tested the conjunction—rather than the global—null hypothesis, allowing us to infer a conjunction of two effects (risky versus safe in BART and monetary gambles) at significant voxels (Friston et al., 2005). For this purpose, we (1) masked the two contrasts with an inclusive *risk matrix* mask containing bilateral NAcc, AIns, and ACC ROIs (volume = 3,132 voxels), (2) applied a small volume correction at peak-level FWE, *p* < 0.05 to each of the two contrasts, and (3) computed the conjunction (i.e., intersection) of the two masked, small-volume corrected contrasts. To examine whether regions outside of the *risk matrix* showed joint activation increases in the two measures, we computed an exploratory conjunction analysis across the entire brain (peak-level FWE correction, *p* < 0.05). We used the Multi-image Analysis GUI Mango^7^ to visualize group-level activation and their intersection on a standard group template in the MNI space. We report the coordinates of local maxima in the MNI space (mm). Anatomical labels for neural regions were obtained from the Neuromorphometrics atlas implemented in SPM8.

##### Individual differences analyses

Turning to our output models, we performed individual differences analyses to understand the extent to which neural activation in *risk matrix* regions was preserved across measures (RQ2) and significantly linked to risk-related outcomes, including within-session behavior in the two fMRI tasks, as well as out-of-session associations with risk preference factors (RQ3). All individual-differences analyses were conducted for residualized neural and behavioral indices, that is, after regressing out effects of age and gender. Initial plotting of the mean beta values indicated relatively normally distributed mean signals for both measures, except for a small number of possible outliers (Supplementary Figure 2) for signals extracted for the ACC from the BART contrast, as well as for the ACC and AIns from monetary gambles. To estimate the biasing effect of these outliers, we computed robust regression analyses (“rlm” function in R package MASS using method “MM”) (Venables and Ripley, 2002) and computed the correlation between the beta coefficients for the neural predictors. The beta coefficients obtained from standard and robust regression analysis correlated highly (*r* = 0.97, *p* < 0.001), but further inspection of the coefficients suggested meaningful discrepancies for models which included BART ACC activation or monetary gambles ACC and AIns activation. To obtain robust results, we computed and report Spearman rank-order correlation coefficients for all individual-differences analyses.

To control for the number of individual differences analyses, we used FWE correction and specified the following four test families for our within-session ROI analyses: (1) brain–brain associations, (2) brain–behavior associations for the BART (RQ3), (3) brain–behavior associations for monetary gambles, and (4) brain–behavior associations across the two measures. We computed three tests per family, one per ROI (cf. Figure 4), resulting in a corrected significance level of *p* < 0.017. For the out-of-session associations between task activation and the BBRS risk preference factors, we defined six test families (one per ROI/fMRI task contrast) comprising eight tests each. This resulted in a corrected significance level of *p* = 0.05/8 = 0.00625; we report which of the results fall below the FWE-corrected significance threshold.

## Results

### Risk-Taking Behavior in the BART and Monetary Gambles

Initial analyses were carried out to examine if risk-taking behavior elicited by the two fMRI tasks was comparable to behavioral patterns observed in previous studies. Confirming the experimental manipulation of reward balloon capacity in the BART (Figure 2A), the average number of pumps was significantly higher for high-capacity balloons compared to low-capacity balloons (*t*_115_ = 7.28, *p* < 0.001, mean difference = 1.05, Cohen’s *d* = 0.80; see Supplementary Table 1 for additional descriptive statistics). Mixed-effects modeling analyses confirm this aggregate pattern and reveal individual as well as task-specific effects on the decision to take a risk: On a given trial in the BART, the number of pumps was higher for male gender, high-capacity balloons, and no explosion on the previous trial (Supplementary Table 2). There was no overall effect of trial number on pumping behavior. As illustrated by the heatmap in Figure 2B, decisions in monetary gambles were influenced in the expected direction by the magnitude of gains and losses: Accept probabilities were highest for high gain–low loss options (lower right quadrant), lowest for low gain–high loss options, and in between for high gain–high loss and low gain–low loss options (see also Supplementary Table 1 for additional descriptive statistics). Results from the mixed-effects logistic regression model for monetary gambles suggest that acceptance of a risky gamble was more likely for higher participant age, male gender, higher gain, and lower (absolute) loss (Supplementary Table 3).

Overall, the behavioral patterns observed in the BART and monetary gambles are in line with previous findings (Tom et al., 2007; Schonberg et al., 2012; Mamerow et al., 2016; Yu et al., 2016). At the level of the group, the two behavioral indices of interest, i.e., standardized residual mean number of pumps (across balloon capacities) in the BART and the standardized residual proportion of Accept decisions for monetary gambles, were approximately normally distributed (see margins in Figure 2C). The association between mean number of pumps in the BART and the proportion of Accept decisions in monetary gambles was negative and not statistically significantly (*r* = -0.16, *p* = 0.09; Figure 2C). That behavior in the two tasks was not correlated did not result from aggregating the two runs to compute one behavioral index for each measure because risky choice was relatively consistent over the two runs in the BART (*r*_*meanpumps*_ = 0.62, *p* < 0.001) and monetary gambles (*r*_*propaccept*_ = 0.84, *p* < 0.001).

### Group-Level Activation for Risky Versus Safe Decisions in BART and Monetary Gambles

To address RQ1, we first computed voxel-wise whole-brain group-level task activations for each measure separately in order to (1) examine if the current implementation of the BART and monetary gambles resulted in group-level task activations comparable with previous research and (2) understand the neural basis on which we performed all subsequent analyses. Initial analyses for the BART yielded no group-level activation differences for the contrast “Pumps reward > Pumps control” between low (12) and high (20) capacity balloons [whole-brain peak-level family-wise error correction (FWE), *p* > 0.05]; as a result, we collapsed events across capacity. For the BART, contrasting pumps on reward balloons with pumps on control balloons revealed several areas of widespread activation differences (whole-brain peak-level FWE, *p* < 0.05) including increased activation for pumping on reward relative to control balloons in a large cortical cluster (*k* = 43,995) encompassing the bilateral supplementary motor cortex, cingulate cortex, and middle segment of the superior frontal gyrus and anterior insula (Supplementary Table 4 for peak coordinates and test statistics). Plotting of this contrast also highlighted the striatum as an area of increased activation (Figure 3A, red–yellow gradient). We also observed several large voxel clusters indicating decreased activation for pumping on reward relative to control balloons (Supplementary Table 4), including a large cluster (*k* = 38,440) spanning the angular gyrus, precuneus, and posterior insula, as well as clusters in medial frontal cortex and lateral orbital gyrus (Figure 3A, blue–green gradient). For monetary gambles, contrasting accept with reject decisions revealed increased activation (“Accept > Reject”) in the striatum (caudate), angular gyrus, and inferior occipital gyrus but no reverse (“Accept < Reject”) effects (whole-brain peak-level FWE, *p* < 0.05) (Figure 3B and Supplementary Table 4). Overall, the whole-brain group-level activations associated with risky choice in the BART and monetary gambles in this study reflect group-level results reported in previous studies (Tom et al., 2007; Rao et al., 2008; Schonberg et al., 2012; Barkley-Levenson et al., 2013; Canessa et al., 2013; Pletzer and Ortner, 2016; Yu et al., 2016; Kohno et al., 2017).

### Conjunction of Group-Level Activation Between Tasks

On the basis of our group-level analyses suggesting activation to be predominantly observed for contrasts targeting increased activation for risky versus safe options (Figures 3A,B), we investigated the intersection of the contrasts “Pumps reward > Pumps control” in the BART and “Accept > Reject.” Restricting our analyses to *risk matrix* regions (Figure 3C), a conjunction analysis of group-level activation in the BART and in monetary gambles revealed a common, locally restricted signal in the NAcc (small volume correction, peak-level FWE, *p* < 0.05; Figure 3D, orange region), as well as a very small region in ACC. Referring back to RQ1, opting for the risky option thus seems to elicit group-level measure-invariant neural signals in NAcc and partly ACC but not the insula. Exploratory analyses outside of *risk matrix* regions revealed a more extensive overlap of increased activation, especially in cortical areas and striatum (whole-brain peak-level FWE, *p* < 0.05; Figure 3D, light orange regions). Next, we conducted individual-level analyses to investigate whether neural function associated with risky choice in *risk matrix* regions was preserved across the two fMRI tasks and to examine their explanatory power within and out of measure, as well as within and out of session.

### Correlations Between Tasks in Canonical Activations

In the first set of within-session analyses targeting RQ2, we assessed whether activation associated with risky versus safe decisions in *risk matrix* regions was preserved (i.e., positively correlated) across the two fMRI tasks. Although behaviorally the two measures did not correlate, it is nevertheless possible that activation in individual neural regions shows (relatively) higher convergence, for instance as a function of shared cognitive or affective components. As illustrated by the scatterplots in Figure 4A, we obtained mixed results. NAcc activation in the BART was positively associated with NAcc activation in monetary gambles (ρ = 0.22, *p* = 0.02), but this result was insignificant after FWE correction (*p* < 0.017). Correlation coefficients for mean activation in ACC and AIns were negative and not significant; that is, mean activation was not preserved between measures (ρ = -0.14, *p* = 0.14; ρ = -0.17, *p* = 0.08, respectively). The overall pattern of results was not influenced by BART contrast analysis (Supplementary Section 1.3 and Supplementary Figure 3). In summary, our analyses to address RQ2 suggest that, although at group level the two measures converged (albeit with limited spatial extent), individual differences were not (significantly) positively correlated across measures: we found some group- but not individual-level consistency for experience- and description-based risk taking (Bornstein et al., 2017).

### Correlations Between Task Activation and Task Behavior

In a second set of within-session analyses targeting RQ3, we examined whether activation in *risk matrix* regions was associated with behavior, both within measure (i.e., with neural and behavioral indices originating from the same fMRI task) and out of measure (i.e., neural index from one fMRI task and behavioral index from the other fMRI task). In particular, we examined the extent to which neural indices extracted from (1) BART were associated with mean number of pumps (within measure), (2) monetary gambles were associated with proportion of accepted gambles (within measure), and (3) BART were associated with proportion of accepted gambles in monetary gambles (out of measure). Given the temporal order of the two measures, we did not test whether neural signal in monetary gambles accounted for BART behavior.

As shown in Figure 4B, we obtained significant (after FWE correction, *p* < 0.017) negative associations for the BART between the mean number of pumps and mean activation in ACC (ρ = -0.26, *p* < 0.01) and NAcc (ρ = -0.24, *p* = 0.01) but not AIns (ρ = -0.2, *p* = 0.03). For monetary gambles (Figure 4C), ROI analyses revealed significant (after FWE correction) within-task negative associations between the proportion of Accept decisions and neural activation in ACC (ρ = -0.37, *p* < 0.001), AIns (ρ = -0.52, *p* < 0.001), and NAcc (ρ = -0.42, *p* < 0.001). To put these findings into context, the within-task associations between risky choice and ACC activation obtained for both the BART and monetary gambles are in line with expectations based on the *risk matrix* framework, as is the direction of associations based on insula activation. The observed negative associations between NAcc and risky choice confirm some previous findings (Pletzer and Ortner, 2016; Büchel et al., 2017) but run counter to others (Courtney et al., 2018; MacNiven et al., 2018); we return to this dissociation in the discussion. For the final set of within-session analyses, we observed no significant out-of-measure associations between the proportion of Accept decisions in monetary gambles and BART activation in ACC (ρ = 0.07, *p* = 0.47), AIns (ρ = 0.02, *p* = 0.79), or NAcc (ρ = -0.03, *p* = 0.76) (Figure 4D).

To summarize, analyses targeting RQ2 suggest that region-specific risk-related activation was not or only marginally preserved between the BART and monetary gambles, suggesting measure-specific processing at the level of the individual. Moreover, analyses targeting the within-session aspect of RQ3 suggest that, although the current neural markers associated with risky choice can account for some behavior within fMRI task, they do not manage to cross a vital barrier for prediction and ultimately application, namely, out-of-measure associations. One possible explanation for this pattern could be our choice of outcome, that is, the use of summary behavioral indices stemming from a single fMRI task. To address this potential shortcoming, we investigated the out-of-session explanatory power of neural indices for psychometrically derived risk preference factors.

### Correlations Between Task Activation and Risk Preference Factors

**FIGURE 5:**
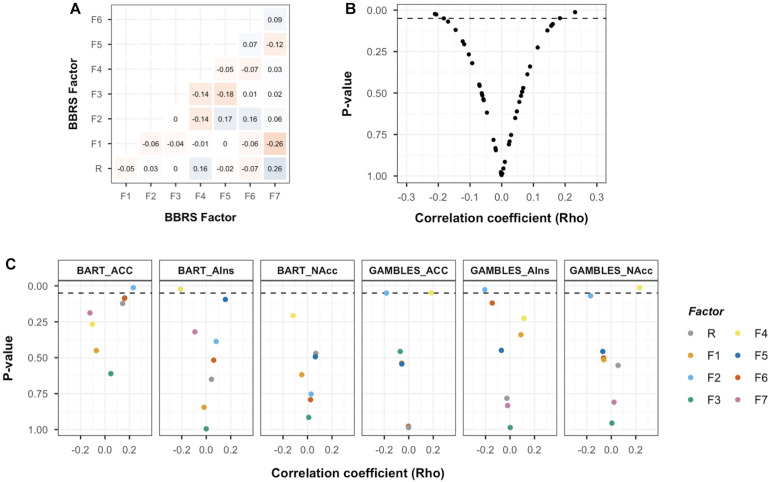
Basel–Berlin Risk Study (BBRS) risk preference factors and association with neural markers of risky choice. **(A)** Pearson correlation coefficients for associations between risk preference factors in the BBRS imaging subsample (*N* = 116). **(B)** Scatterplot for correlation coefficients and associated *p*-values for brain–behavior associations. Dashed line represents significance level of *p* < 0.05 (uncorrected for multiple testing). **(C)** Scatterplot for correlation coefficients and associated *p* values for all brain–behavior associations, organized by region of interest (ROI) and colored by risk preference factor. Dashed line represents significance level of *p* < 0.05. Balloon Analog Risk Task (BART) mean activation extracted from “Pumps reward > Pumps control” contrast; GAMBLES mean activation extracted from “Accept > Reject” contrast; ACC, anterior cingulate cortex; AIns, anterior insula; NAcc, nucleus accumbens; R, general risk-preference factor; F1, health risk taking; F2, financial risk taking; F3, recreational risk taking; F4, impulsivity; F5, traffic risk taking; F6, occupational risk taking; F7, choices among (monetary) lotteries.

Pearson correlation coefficients between the psychometric risk preference factors (Figure 5A) were comparable to the correlation coefficients observed in the full BBRS sample (Frey et al., 2017). The main analysis to address the between-session aspect of RQ3 involved a set of Spearman correlation analyses between the risk preference factors and the neural markers extracted from three ROIs for each of the two measures’ main contrast. All analyses were performed on residuals (i.e., after regressing out effects of age and gender). As illustrated in Figure 5B, the majority of analyses between neural markers associated with risky choice and risk preference factors resulted in non-significant (*p* > 0.05) correlation coefficients close to 0 (mean_*rho*_ = 0.001, median = -0.001, range = -0.21–0.23; Supplementary Figure 4). Separating these results by factor and ROI, we observed a pattern of notable exceptions: the domain-specific risk preference factors, F2 (financial risk taking) and F4 (impulsivity) consistently returned correlation coefficients with the highest (absolute) magnitude for neural markers from both BART and monetary gambles (Figure 5C; see Supplementary Figure 5 for results from additional BART contrast analyses). Interestingly, the directionality of associations differed for the two tasks. For monetary gambles, financial risk taking correlated negatively with risk-related activation in NAcc (ρ = -0.17, *p* = 0.069), ACC (ρ = -0.18, *p* = 0.05) and AIns (ρ = -0.21, *p* = 0.03), whereas impulsivity correlated positively with risk-related activation in NAcc (ρ = 0.23, *p* = 0.02) and ACC (ρ = 0.18, *p* = 0.049). For the BART, risk-related activation in ACC correlated positively with financial risk taking (ρ = 0.23, *p* = 0.013), and AIns activation correlated negatively with impulsivity (ρ = -0.21, *p* = 0.023). However, none of these associations survive FWE corrections (all *p* > 0.00625). We turn to the implications of the current results in the discussion.

## Discussion

In this study, we examined the role of the measures used to capture neural and behavioral individual differences in risk taking by focusing on associations between risk-related neural activation and various indices of (real life) risk taking. Based on two popular behavioral measures of risk taking—the BART and monetary gambles—our results suggest limited functional convergence in neural risk matrix regions at both group and individual levels, on the one hand, and mixed results concerning brain–behavior associations for single performance indices as well as for psychometrically derived risk preference factors on the other. That different measures of risk taking elicit different choices has previously been discussed, both at the level of behavior (Mata et al., 2011; Mamerow et al., 2016; Rosenbaum et al., 2018) and their neural correlates (FitzGerald et al., 2010; Congdon et al., 2013; Pletzer and Ortner, 2016). Indeed, the (behavioral) differences observed across the life span between measures of experienced and described risk (of which the BART and monetary gambles are respective examples) have led to the proposition of an affect-based model of adolescent risk taking (Rosenbaum et al., 2018). However, very few empirical studies have benefited from some of the major strengths of our work, including a large sample size, within-participant design, *a priori* defined ROIs, and a mixture of group and individual difference analyses aimed specifically at addressing issues of convergent validity and explanatory power (i.e., brain–behavior associations) within and across behavioral measures of risk taking. A further main strength of this study is its link to the BBRS, which allowed us to use latent variables capturing the psychometric structure of risk preference as outcome variables for brain–behavior associations.

Mirroring previous reports of joint group-level activation increases in bilateral striatal regions for risky versus safe options in the BART and a Game of Dice task (Pletzer and Ortner, 2016), our conjunction results in NAcc support the notion of a measure-invariant core neural signal of choice across risk-taking measures (Knutson and Huettel, 2015). The striatum in general is a central structure implicated in reward processing (Izuma et al., 2008; Haber and Knutson, 2010); to the extent that risk taking is driven by the motivation to achieve a reward (Ravert et al., 2019), striatal activation is a common neural correlate of risk taking (Knutson and Huettel, 2015). However, it has been suggested that attention plays an important role for activation especially in the ventral striatum (and thus to a large extent the NAcc), perhaps even independently of the anticipated rewards (Bjork, 2020). Thus, although our contrast analyses for BART and monetary gambles both resulted in increased activation in the NAcc, the underlying source of the signal may be differentially driven by attentional demands. Moreover, the ventral striatum is also implicated in the coding of prediction error (Hare et al., 2008), which may suggest a functional convergence on monitoring of the *status quo*. Unfortunately, the two fMRI tasks used here do not allow us to disentangle these different signal sources. However, to observe a conjunction of increased NAcc activation for the two risk-taking measures at the level of the group despite the many differences between the two measures is encouraging.

The observed group-level activation in AIns for the BART, but not monetary gambles, supports the argument that experience-based measures involve potentially more affective and motivational processes compared with description-based measures (Hertwig and Erev, 2009; Schonberg et al., 2011; Rosenbaum et al., 2018). The AIns is heavily implicated in signaling subjective feelings and explicit motivation (Namkung et al., 2017) and is thought to inhibit risky choice (Knutson and Huettel, 2015). In this study, the BART, but not monetary gambles, involved choice feedback, which may have led to the observed neural dissociation in AIns. Indeed, the insular cortex was a further source of common activation in previous comparative work, where both the experience- and description-based measures included feedback between choices (Pletzer and Ortner, 2016). Thus, while there may be core regions associated with risk preference, some, like the NAcc, may be more core than others, depending on the measure used. Crucially, the limited regional overlap of group-level activation patterns reported here and previously (Pletzer and Ortner, 2016) is not an artifact of the contrast analysis targeting a summary decision signal for risky versus safe decisions; similar findings were obtained for more targeted components such as value or the probability of incurring a loss (FitzGerald et al., 2010).

Past work has made clear that group averages are not necessarily reflective of individual-level behavioral (Blanco et al., 2011; Bornstein et al., 2017; Fisher et al., 2018) or neural patterns (Fliessbach et al., 2010; Elliott et al., 2020). We found that a group-level activation increase for risky versus safe decisions was localized in the NAcc for both fMRI tasks, yet NAcc, AIns, and ACC activation was not (significantly) positively correlated between the two tasks. While the fMRI tasks used here differed on many aspects aside from whether risk was experienced or described, previous research (FitzGerald et al., 2010) with carefully matched task implementations has shown that outcome probability correlated more strongly with activity in ACC for experienced but AIns for described risk. In other words, the same choice component can indeed be represented differently as a function of the task. One explanation may be found in the low test–retest reliability of fMRI signal elicited with reward-based decision making tasks (Elliott et al., 2020); a highly variable brain signal creates a bottleneck for the extent to which activations from different tasks correlate within subject (or with external variables), even in canonical risk-taking regions like NAcc, AIns, and ACC.

Turning to our brain–behavior associations, it has been suggested that experience-based risk-taking measures such as the BART are ecologically more valid exactly because the sequential nature and exposure to outcomes elicits stronger affective responses (Lejuez et al., 2002; Schonberg et al., 2011; Rosenbaum et al., 2018). Whether this translates into stronger brain–behavior associations, however, is an open empirical question. Although our results revealed significant brain–behavior associations within fMRI task for both BART and monetary gambles, we observed larger effect sizes [i.e., higher (absolute) correlation coefficients] for activation extracted from monetary gambles compared with BART. Importantly, our supplementary analyses suggest that our findings are not tied to a particular BART contrast analysis. Instead, the observed differences in brain–behavior associations may arise as a result of varying task demands (Figner et al., 2009; Hertwig and Erev, 2009; Mata et al., 2011; Mamerow et al., 2016; Rosenbaum et al., 2018). For example, if the sequential (feedback-based) aspect of the BART involves more attention than the descriptive aspect of monetary gambles, and if attention can drive signal in the ventral striatum, perhaps even independently of anticipated rewards (Bjork, 2020), then one hypothesis may be that the contribution of attentional demand to NAcc signal attenuates brain–behavior associations for external variables that assume a link based on reward sensitivity. Pinning down the mechanisms that give rise to NAcc signal in different tasks will directly inform our expectations for brain–behavior associations.

Furthermore, the reliability of the brain signal itself may also attenuate brain–behavior associations (Elliott et al., 2020). For example, reliable group- and individual-level risk-related activation has been reported for the BART, especially in canonical regions (Korucuoglu et al., 2020; Li et al., 2020). However, the reported reliability estimates are based on BART implementations that included only one type of balloon, and as our behavioral analyses suggested, balloon capacity affects risk-taking behavior. Thus, certain BART implementations may reduce test–retest reliability, which in turn constrains the extent to which strong brain–behavior associations can be expected.

Concerning the directionality of associations between risk-related neural function and outcomes, our negative brain–behavior associations for AIns and ACC are in line with proposed functional dissociations, whereby more affect-based inhibition and control-related processes are associated with less risk taking (Knutson and Huettel, 2015; Pletzer and Ortner, 2016). The observed negative association(s) between NAcc activation and risky choice within task mirror(s) previous results suggestive of a compensatory link between hyporesponsiveness of dopamine-modulated brain regions in the reward circuit and risk-related or impulsive behaviors (Pletzer and Ortner, 2016; Büchel et al., 2017). However, existing studies that have focused on brain–behavior associations for (reward and/or risk-related) NAcc activation have reported both positive and negative associations (Sherman et al., 2018; Bjork, 2020). For neural markers elicited with the same fMRI task (Büchel et al., 2017; Courtney et al., 2018; MacNiven et al., 2018), different directionalities may provide candidate mechanisms for how relative NAcc activation in anticipation of reward from generic (e.g., points, money) versus phenotype-specific (e.g., drug paraphernalia) cues underpin the initiation and trajectory of pathological risk taking (e.g., substance use). In contrast, for neural markers elicited with different fMRI tasks, opposing directionality for brain–behavior associations may harbor effects of task-specific parameters, including attentional demands, the presence of ambiguity, or the exact loss function (Congdon et al., 2013; Sherman et al., 2018; Bjork, 2020).

Interestingly, brain–behavior associations for specific kinds of risk taking, as operationalized by the domain-specific risk preference factors, revealed the highest absolute (albeit non-significant after FWE correction) correlations for the factors F2 (financial risk taking) and F4 (impulsivity) but with differing directions for BART and monetary gambles. The negative associations between NAcc, AIns, and ACC activation in monetary gambles and the proportion of Accept decisions (within task) as well as F2 (out of task) may point toward the monetary aspect of both outcome variables as a potential mechanism, yet we did not observe similar associations with F7 (choices in monetary lotteries). In turn, the negative association between F4 and BART AIns activation follows research highlighting the role of the AIns for incentivized inhibition (Leong et al., 2018), yet at the same time underlines the role of the task considering that we found no association between AIns activation in monetary gambles and F4. To the extent that the BART assesses impulsive risk taking, and description-based gambles tap more into reflective risk taking (Pletzer and Ortner, 2016), these findings provide informative starting points for future research on phenotypes with strong links to impulsivity, such as addiction (Verdejo-García et al., 2008).

Our study has some limitations worth addressing. First, we analyzed data from two fMRI tasks as prototypical examples of experienced and described risk, limiting generalization. However, research suggests that other risk-taking measures do not fare much better regarding behavioral consistency (Frey et al., 2017), thus would probably not yield higher convergence at group or individual level. Implementing additional measures based on experienced (Bechara et al., 1994; Figner et al., 2009) and described risk (Holt and Laury, 2002) could nevertheless address questions such as whether the convergence of measures is overall higher for experienced or described risk and within the respective classes of experienced versus described risk. On a related note, implementations of decision-theoretic models of risky choice do not fare much better with regard to convergence of computationally derived parameters (Pedroni et al., 2017); thus, we do not expect the overall pattern of results to be substantially different for latent performance-related outcome variables instead of the simpler behavioral indices used here for BART and monetary gambles.

Second, our results do not speak to the neural correlates of specific decision components or motivational factors, including reward, uncertainty, variance, or probability (of incurring a loss). In part, this is a limitation for contrast analyses that average activation over particular events (e.g., *Pump* or *Accept* decisions), and one way to disentangle different components is to use parametric analyses that map neural activation to specific functional forms, such as increases in reward or probability of loss. However, standard implementations of the BART, such as the one used here and elsewhere (Schonberg et al., 2012; Helfinstein et al., 2014; Braams et al., 2015), do not allow for the isolation of these signals even with parametric analyses because reward and probability of loss increase monotonically over a given trial, that is, many individually contributing decision components are confounded. However, it should also be noted that reward and the probability of loss are correlated in many real-world domains (Pleskac and Hertwig, 2014). Similarly, the current implementation of monetary gambles, although following on from previous studies (Tom et al., 2007; Barkley-Levenson et al., 2013), is unsuitable for investigating the effect of probability (because this was kept constant across trials) or dissecting the effects of choice-motivating aspects such as variance, expected value, gains, and losses (because these are confounded). Whether disentangling and comparing specific choice-related components (e.g., value, probability of loss) achieves a more successful mapping of the neural correlates of risk taking between different measures, and also whether this would result in successful out-of-measure brain–behavior associations, is an empirical question.

A third and related limitation stems from the temporal characteristics of the two measures used. The current implementations do not allow us to incontrovertibly dissociate different decision stages, such as the separation of reward anticipation and decision. Previous studies have focused predominantly on neural signals associated with the anticipation of rewards, showing the signal’s potential for prediction (Büchel et al., 2017; Courtney et al., 2018; MacNiven et al., 2018; Krönke et al., 2020). However, other studies have focused on the neural signal associated with actual choice or receipt of an outcome, also showing potential of the signal for explaining individual differences in risk and ambiguity preferences (Blankenstein et al., 2018). Returning to the point of understanding our measures better, it would be exceptionally useful to make more principled decisions about which measure to use and which decision stage for a given measure isolates the most informative neural signal for brain–behavior associations.

Fourthly, our design is prey to order effects because we opted for a fixed task order. Randomization would have required splitting the sample into two groups based on order, thus reducing power; however, risky choice was relatively consistent across the two runs for each measure, plus the overall level of observed risk taking in the BART and monetary gambles as well as group-level neural activation was comparable to previous independent investigations (Tom et al., 2007; Schonberg et al., 2012). Taken together, our behavioral and neural results for the individual measures do not indicate major order effects.

Many longitudinal, clinical, and developmental research designs focus on risk preference as a critical predictor or outcome, often aiming to establish links between individual differences in risk preference and neural function (Moffitt et al., 2011; Braams et al., 2015; Büchel et al., 2017; Casey et al., 2018). Such endeavors are undoubtedly commendable and have the scope to be hugely beneficial for individuals and societies, but it is currently unclear to what extent previous results vary as a function of the measures used to operationalize risk taking and indeed what the best operationalizations are to isolate (neural) targets for prediction. To be clear, we *do not* advocate that neural markers have no predictive validity, in general or for risk preference, neither do we ignore the complexity of the phenotype under investigation. We simply wish to highlight the challenges ahead and open the discourse by empirically comparing two widely and often interchangeably used risk-taking measures, in particular their potential for brain–behavior associations.

Based on our results, we make the following recommendation for future work. First and foremost, we are hopeful that our findings motivate further research into the nature and assessment of risk preference, risk-taking behavior, and their neural underpinnings. We still know comparatively little about how the brain integrates different aspects of a decision situation under uncertainty, especially when this is laden with incidental or contextual variables such as affect or prior experience (Knutson and Huettel, 2015; Samanez-Larkin and Knutson, 2015). To this end, it may be indispensable to develop novel and representative behavioral measures (Steiner and Frey, 2020) that facilitate the disentangling of risk-related cognitive and affective processes. Second, the assumption of generalizability from group to individuals is often not backed up by empirical evidence, posing a threat for individual studies and also research involving human subjects in general (Fisher et al., 2018). To successfully target individual differences in risk taking and understand the biological underpinnings, a switch is required—especially within neuroscience—from group- to individual-level research (Foulkes and Blakemore, 2018; Rosenberg et al., 2018) and from single- to multimeasure research (Poldrack et al., 2018). As a third and final recommendation, we call for greater transparency in the reporting of how and why risk-taking measures were selected for a given research study. This will not only lead to more principled decisions during the research design stage but hopefully push the research community toward establishing a much-needed taxonomy of measures and their core biological underpinnings. Regrettably, at present, we cannot formally compare our risk-taking measures because despite promising attempts to bring order to measurement chaos (Mata et al., 2011; Poldrack et al., 2011; Frey et al., 2017; Pedroni et al., 2017; Eisenberg et al., 2019), we still lack a clear understanding of the data-generating processes involved. At a time when the reliability and replicability of neuroimaging research has come under scrutiny (Botvinik-Nezer et al., 2020; Elliott et al., 2020), tackling these open issues is crucial for the interpretation and generalizability of studies linking neural correlates with risk-taking-related developmental trajectories (Moffitt et al., 2011; Braams et al., 2015; Casey et al., 2018; Rosenbaum et al., 2018) and clinical outcomes (Büchel et al., 2017; MacNiven et al., 2018). If the ultimate aim is to help individuals navigate an uncertain, risk-laden world and make better choices, we first need to navigate and map the mainly uncharted territory of our risk preference measures.

## Data Availability Statement

The datasets presented in this study can be found in online repositories. The names of the repository/repositories and accession number(s) can be found below: Behavioral data, ROI data, and analyses scripts are available via the Open Science Framework (https://osf.io/jrk7y/). Unthresholded group-level statistical parametric maps for fMRI analyses of BART and monetary gambles are accessible via NeuroVault (https://neurovault.org/collections/WLVWHWMC/). A preprint of this manuscript was deposited on PsyArXiv (https://psyarxiv.com/3sc9j/).

## Ethics Statement

The studies involving human participants were reviewed and approved by the German Society for Psychology, and the Ethics Committee of the Max Planck Institute for Human Development. The patients/participants provided their written informed consent to participate in this study.

## Author Contributions

RM, DO, AH, JR, RF, AP, and RH designed the research. LT, LH, and RM acquired the neuroimaging data. LT and AH analyzed the neuroimaging data. LT analyzed the behavioral data. RF and AP analyzed the laboratory data and extracted the psychometric risk-preference factors. LT, RM, RF, AH, FB, and DO interpreted the results. LT drafted the manuscript. AH, DO, LH, FB, JR, AP, and RH edited the manuscript. LT, RF, and RM wrote the final version of the manuscript. All authors contributed to the article and approved the submitted version.

## Conflict of Interest

The authors declare that the research was conducted in the absence of any commercial or financial relationships that could be construed as a potential conflict of interest.

## Footnotes

1 neurosynth.org

2 http://r-project.org

3 http://CRAN.R-project.org/package=lme4

4 http://CRAN.R-project.org/package=lmerTest

5 http://www.fil.ion.ucl.ac.uk/spm/software/spm8/

6 www.brain-development.org

7 http://rii.uthscsa.edu/mango/index.html
