# Supplementary material for: Brain–Behavior Associations for Risk Taking Depend on the Measures Used to Capture Individual Differences
Source: Front Behav Neurosci. 2020 Nov 17;14:587152. doi: 10.3389/fnbeh.2020.587152 (PMC7705248; doi:10.3389/fnbeh.2020.587152)
Supplement: Supplementary file 1 [file Data_Sheet_1.PDF]

Supplementary Material

1 Supplementary Information

1.1 Supplementary information concerning fMRI data preprocessing

Preprocessing of the fMRI data involved realignment and co-registration of functional to structural volumes. Volumes were nonlinearly warped into standard stereotactic (MNI) space based on structural scans using the New Segment method (Ashburner and Friston 2005). To control for spatial noise and average effects that may arise as a function of residual anatomical differences between subjects, images were spatially smoothed using an 8-mm full-width half-maximum Gaussian kernel. After preprocessing, we conducted two sets of analyses: a set of input models to estimate voxel-wise activation differences for specific risk-taking related events, and a set of output models in which we link the estimated (mean) activation differences to risk-related outcomes. We describe each of these in turn, starting with the specification of the input models.

1.2 Supplementary information concerning GLM weights for the main contrast analyses

To estimate the relative differences in activation differences between the events of interest in the BART, we specified the following contrast weights: [-2 0 1 0 1 0 0 0 0 0 0 0 0 0] to estimate 'Pumps reward > Pumps control', [2 0 -1 0 -1 0 0 0 0 0 0 0 0 0] to estimate 'Pumps reward < Pumps control', [0 0 1 0 -1 0 0 0 0 0 0 0 0 0] to estimate 'Pumps low capacity balloons > Pumps high capacity balloons', and [0 0 -1 0 1 0 0 0 0 0 0 0 0 0] to estimate 'Pumps low capacity balloons < Pumps high capacity balloons'. For mixed gambles, we emulated previous analyses (Barkley-Levenson, Van Leijenhorst, and Galván 2013), in order to facilitate a contrast analysis that is comparable for risk in both the BART and monetary gambles. Specifically, that means we contrasted individuals' *Accept* decisions with *Reject* decisions. To estimate activation differences for risky versus safe decisions in monetary gambles, we used the contrast weights [1 -1 0 0 0 0 0 0] to estimate *Accept* > *Reject*, and [-1 1 0 0 0 0 0 0] to estimate *Accept* < *Reject*.

1.3 Additional analytic approaches to BART fMRI contrast analyses

The BART is a dynamic decision-making measure that has found widespread application in both behavioral and neuroimaging studies (Schonberg et al. 2012; Rao et al. 2008; Helfinstein et al. 2014; Lejuez et al. 2002). However, although the dynamic nature is thought to increase the ecological validity of this measure (Schonberg, Fox, and Poldrack 2011; Lejuez et al. 2002), this poses challenges for modeling behavior and estimating neural activation differences because decision components such as risk (i.e., probability of incurring a loss), reward and loss magnitude or positively correlated. As such, it may not be possible to completely isolate these components (and their neural correlates) in the BART. Moreover, different contrasts have been proposed to target different components of risky choice (Schonberg et al. 2012; Rao et al. 2008). For example, a standard BART analysis contrasts pumps on reward balloons and pumps on control balloons, but aggregates across pumps on reward balloons. As a result, this contrast misses the rising tension (i.e., risk, that is, the probability of incurring a loss) associated with additional pumping. To capture this

more affective aspect, an alternative contrast targets the neural parametric response to increased pumping on a given reward balloon as compared with a control balloon. However, contrasts between reward and control balloons are contrasts between decisions made under risk versus no decision making, which may misattribute more general decision-related processes. A contrast between decisions to actively take a risk (pumping) or not (stopping, i.e. cash out) may resolve this issue.

Considering the ubiquity of the BART and the use of different contrast analyses, we performed supplementary analyses for mean activation differences extracted from these additional two contrasts in order to gauge variability in effect size as a function of the BART contrast analysis. For instance, the parametric contrast is thought to capture the rising tension, and thus may capture insula activation differences more succinctly than contrasts that ignore pump history. As such, brain-behavior associations with an effective link may be stronger for contrast analyses better suited to capture affective neural processes.

For the parametric analysis, we contrasted the parametric regressors for onset vectors from the main general linear model as follows: [0 -2 0 1 0 1 0 0 0 0 0 0 0 0] to estimate 'Parametric pumps reward > Parametric pumps control', and [0 2 0 -1 0 -1 0 0 0 0 0 0 0 0] to estimate 'Parametric pumps reward < Parametric pumps control' (see Supplementary Figure 1A for example individual-level design matrix). For the contrast of pumps on reward balloons versus cash-out decisions, we specified a separate general linear model based on the following regressors (in that order): Onset vector of pumps for control balloons, two onset vectors for pumps on reward balloons, onset vector for cash outs, onset vector for explosions, and six motion parameters estimated during the realignment process. Our main analysis focused on contrasting pumping on risky balloons with the decision to cash out, but one potential confound of such a contrast is that there may be systematic biases in the history of trials leading to a cash out decision. Concretely, cash-out decisions may not be similarly distributed across trials with regards to their onset, but perhaps happen early on in the trial as a result of mounting tension or the motivation to ensure some saved earnings. In building a contrast between cash-out events and pump events, any such systematic biases may also bias the neural signals. To address this issue, for every individual included in the analysis, we isolated the maximum serial position of all cash-out decisions and used this to cap the serial position of pumps included in the first onset vector, while onsets for the remaining pumps (i.e., those exceeding the maximum serial position of all cash-out decisions) were included in the second pumps vector. The inclusion of additional regressors was intended to explain additional variance, thus returning a 'cleaner' neural signal for the events of interest. We contrasted matched pumps with cash-out decisions, using the contrast weights [0 1 0 -1 0 0 0 0 0 0 0] to assess *Pumps (matched) > Cash out*, and [0 -1 0 1 0 0 0 0 0 0 0] for *Pumps (matched) < Cash out* (Supplementary Figure 1C for example individual-level design matrix). For the two supplementary contrast analyses, we used the same extraction and analysis procedures as described for the main analyses.

In a first step, we ascertained the similarity between neural indices extracted from the different contrast analyses via correlation analyses, with mixed results concerning directionality and magnitude of associations (Supplementary Figure 3B). These initial patterns indicated that neural signal in *risk matrix* regions varies as a function of the BART contrast used. Interestingly, there was much less variability with regards to the overall pattern of brain-behavior associations as a function of the contrast analysis used (Supplementary Figure 3C), in particular for out-of-measure effect sizes (Supplementary Figure 5). This suggests that the BART, regardless of the contrast analysis used, may

not be an optimal behavioral task to capture neural mechanisms that can be reliably associated with risk taking.

1.4 Anterior insula ROI

For neural structures with substantial intra-regional functional and structural variation, extraction of a mean signal from the entire structure may hide functionally important differences between sub-regions. Based on differential cytoarchitecture, connectivity, physiological function and role in pathology, many studies have suggested the insula to be subdivided into an anterior and a posterior region (Namkung, Kim, and Sawa 2017). Indeed, the *risk matrix* framework (Knutson and Huettel 2015) specifically addresses the importance of the anterior insular cortex for decisions made under risk. To restrict our analyses to the anterior aspect of the insular cortex, we constructed the AIns ROI based on previous research (Chang et al. 2013), in which a parcellation of the insular cortex was reported based on a meta-analytic approach to studies examining insula functional connectivity at rest. From this approach the authors derived an anterior-posterior division (posterior cluster center coordinate -38 / -10 / 6), but also revealed an additional division of the anterior region into a dorsal (-38 / 12 / -2) and a ventral (-34 / 8 / -8) cluster. Although we were mainly interested in the anterior-posterior division, we initially set out to visualize these three ROIs to gauge their position and extent. Chang and colleagues (2013) did not provide images that could be used to extract mean signal from the whole of the parcellated sub-regions, thus we constructed spherical ROIs around the MNI-space cluster center coordinates reported above, using the MarsBaR toolbox for SPM8. As we had no a priori hypothesis regarding hemispheric differences, we projected the coordinates reported for the left hemisphere into the right hemisphere by mirroring the coordinates on the x-axis (i.e. dorsal anterior: 38 / 12 / -2, ventral anterior: 34 / 8 / -8, and posterior insula: 38 / -10 / 6).

The radius for the ROI spheres was determined based on inspection of the structural borders of the insula as provided in the Harvard-Oxford cortical structural atlas. We selected the Harvard-Oxford cortical structural atlas as the template for constructing our spheres for two reasons. First, because this was the atlas used in (Chang et al. 2013) to determine the insula ROI, hence we were able to map and visualize our coordinate-based ROIs against the insula mask from which they were derived. Second, because the Harvard-Oxford cortical structural atlas is a probabilistic atlas, thus allowing us to visualize the extent to which differing spheres cover voxels assigned to the insula with different probabilities. The inspection of varying spheres around the three cluster coordinates against the insula structure as provided in the Harvard-Oxford cortical structural atlas was guided by three criteria for selection of the final ROIs: 1) no voxels outside of the boundary of the insula, 2) no overlap between voxels contained in the three spheres, and 3) capture of as many voxels inside the insula as possible given criteria 1 and 2. This process revealed that retaining all three ROIs was inconsistent with our selection criteria, predominantly because of the close proximity of the dorsal and ventral anterior insula clusters resulting in substantially overlapping ROIs. Given the main focus of our analyses on the anterior insula, the AIns ROI was built as a maximally large sphere around the dorsal cluster center coordinate, as this was the more central coordinate and thus captured voxels contained in the ventral anterior cluster. The final ROIs used were a left and a right AIns ROI (center = +-38 / 12 / -2, radius = 7mm, k = 179 voxels).

2 Supplementary Figures and Tables

2.1 Supplementary Tables

Supplementary Table 1. Descriptive statistics for outcome measures for the BART and monetary gambles.

Outcome	Mean (SD)	Range
**BART**		
Number of completed trials (including controls)	60.72 (6.23)	37–73
Number of low-capacity balloons (max. 12)	20.12 (2.12)	12–25
Number of high-capacity balloons (max. 20)	20.25 (2.20)	12–25
Average pumps on low-capacity balloons (max. 12)	4.45 (1.06)	2.40–6.95
Average pumps on high-capacity balloons (max. 20)	5.50 (1.52)	2.25–9.93
Number of explosions experienced	15.81 (3.81)	6–24
Average reaction time pumps control (seconds)	0.63 (0.20)	0.32-1.44
Average reaction time pumps reward (seconds)	0.71 (0.26)	0.33–1.99
Average reaction time cash out (seconds)	0.93 (0.43)	0.38–3.35
**MONETARY GAMBLES**		
Number of valid responses	142.67 (1.96)	133–144
Proportion accepted gambles	0.47 (0.16)	0.13–0.92
Average reaction time *Accept* decisions (seconds)	1.31 (0.47)	0.46–2.98
Average reaction time *Reject* decisions (seconds)	1.30 (0.44)	0.07–2.99

Supplementary Table 2. Fixed-effects standardized regression coefficients from mixed effects linear regression model for trial-by-trial number of pumps in the BART. The outcome variable was number of pumps administered on reward balloons on a given trial. Capacity, explosion on the previous trial, and gender were entered as binary variables, trial number and age were entered as standardized, continuous variables. For categorical variables, we report (in brackets) the level for which the (relative) effect is estimated.

	Estimate	Std. Error	df	t value	p value
Intercept	4.770	0.143	132.672	33.354	<0.001
Age	0.047	0.089	110.427	0.533	0.595
Gender (female)	-0.386	0.177	110.385	-2.177	0.032
Capacity (20)	1.077	0.144	113.169	7.493	<0.001
Trial number	-0.076	0.052	96.362	-1.461	0.147
Previous explosion trial (yes)	-0.309	0.077	111.929	-3.990	<0.001

Supplementary Table 3. Fixed-effects standardized regression coefficients from mixed effects logistic regression model for trial-by-trial decisions in monetary gambles. The outcome variable was individuals' decision to Accept or Reject (0 = Reject, 1 = Accept) a gamble on a given trial. Gain, absolute loss, and age were entered as standardized continuous variables, gender was entered as a binary variable. For the categorical variable, we report (in brackets) the level for which the (relative) effect is estimated.

	Estimate	Std. Error	z value	p value
Intercept	0.070	0.033	2.137	0.033
Age	0.124	0.023	5.398	<0.001
Gender (female)	-0.548	0.046	-11.914	<0.001
Gain	2.030	0.099	20.422	<0.001
Absolute loss	-2.194	0.101	-21.701	<0.001

Supplementary Table 4. Significant peak coordinates obtained from group-level analyses of activation differences in BART and monetary gambles. Presented are peak coordinates obtained from whole-brain contrast analysis of risk taking in the BART (operationalized as pumping on reward balloons versus pumping on control balloons, *'Pumps reward versus Pumps control'*) and monetary gambles (operationalized as acceptance or rejection of a gamble, *'Accept versus Reject'*). Reported are increased and increased activation differences, corrected at peak-level FWE, $p < 0.05$. Coordinates are reported in MNI space (mm). Labels were obtained from the Neuromorphometrics atlas implemented in SPM. Cluster center coordinates are reported in bold font. k = cluster extent in voxels. *Note:* Due to the large extent of some activation clusters, some otherwise salient individual regions are being subsumed under cluster-level labels. Activation cluster plots in the main text (Figure 3) provide a visual guide for neural regions where activation differences for the contrasts of interest were observed.

BART 'Pumps reward > Pumps control'							
Label	*L/R*	*k*	*Peak p (FWE p< 0.05)*	*Peak T*	*x*	*y*	*z*
Supplementary motor cortex	**R**	**43995**	**0.000**	**24.57**	**4**	**22**	**42**
Anterior insula	R		0.000	21.04	40	18	0
Anterior insula	R		0.000	20.88	32	20	-4
Supramarginal gyrus	**R**	**5061**	**0.000**	**17.09**	**46**	**-40**	**46**
Superior parietal lobe	R		0.000	10.97	30	-60	46
Precuneus	R		0.000	10.25	10	-68	48
Occipital pole	**L**	**2518**	**0.000**	**17.04**	**-14**	**-102**	**-2**
Occipital pole	R		0.000	15.34	18	-98	-2
Occipital fusiform gyrus	L		0.000	11.62	-28	-88	-12
Middle temporal gyrus	**R**	**117**	**0.000**	**8.97**	**58**	**-28**	**-16**
BART 'Pumps reward < Pumps control'							
Label	*L/R*	*k*	*Peak p (FWE p< 0.05)*	*Peak T*	*x*	*y*	*z*
Angular gyrus	**L**	**38440**	**0.000**	**21.03**	**-48**	**-70**	**24**
Precuneus	L		0.000	20.02	-6	-56	14
Posterior insula	R		0.000	19.69	38	-14	16
Medial frontal cortex	**L**	**4562**	**0.000**	**18.15**	**-2**	**58**	**-12**
Medial superior frontal gyrus	L		0.000	12.72	-8	64	18
Superior frontal gyrus	L		0.000	11.98	-22	22	44
Lateral orbital gyrus	**L**	**1150**	**0.000**	**15.96**	**-36**	**34**	**-16**
Inferior frontal gyrus (triangular)	L		0.000	14.68	-52	36	2
Inferior frontal gyrus (triangular)	L		0.000	12.96	-52	30	10
Inferior frontal gyrus (triangular)	**R**	**152**	**0.000**	**11.83**	**54**	**38**	**2**
Anterior insula	**L**	**69**	**0.000**	**9.15**	**-34**	**4**	**10**
Central operculum	**R**	**50**	**0.000**	**8.50**	**36**	**6**	**12**
Posterior orbital gyrus	**R**	**42**	**0.000**	**6.94**	**36**	**34**	**-16**
Gambles 'Accept > Reject'							
Label	*L/R*	*k*	*Peak p*	*Peak T*	*x*	*y*	*z*

			(FWE p< 0.05)				
Caudate	**R**	**335**	**0.000**	**8.28**	**10**	**16**	**-2**
Angular gyrus	**L**	**952**	**0.000**	**8.11**	**-32**	**-72**	**36**
Superior parietal lobe	L		0.001	5.88	-28	-64	52
Caudate	**L**	**266**	**0.000**	**7.89**	**-8**	**18**	**-4**
Middle frontal gyrus	**L**	**404**	**0.000**	**7.76**	**-44**	**34**	**14**
Inferior temporal gyrus	**L**	**767**	**0.000**	**7.52**	**-48**	**-64**	**-10**
Inferior temporal gyrus	L		0.000	6.73	-46	-52	-14
Precentral gyrus	**L**	**165**	**0.000**	**6.30**	**-36**	**4**	**26**
Precuneus	**L**	**270**	**0.000**	**6.13**	**-6**	**-58**	**10**
Precuneus	L		0.003	5.54	-16	-60	18
Middle frontal gyrus	**L**	**143**	**0.001**	**5.95**	**-26**	**14**	**50**
Superior frontal gyrus	L		0.004	5.48	-24	30	46
Supramarginal gyrus	**L**	**269**	**0.001**	**5.84**	**-50**	**-38**	**44**
Supramarginal gyrus	L		0.003	5.58	-44	-44	38
Supramarginal gyrus	L		0.023	5.04	-38	-42	44
Posterior cingulate gyrus	**L**	**49**	**0.002**	**5.74**	**-2**	**-34**	**36**
Anterior cingulate gyrus	**R**	**46**	**0.003**	**5.56**	**2**	**38**	**4**
Middle cingulate gyrus	**R**	**9**	**0.010**	**5.27**	**6**	**16**	**18**
Precuneus	**L**	**21**	**0.015**	**5.15**	**-6**	**-48**	**70**
Precuneus	L		0.016	5.13	-4	-56	66
Hippocampus	**L**	**7**	**0.027**	**4.99**	**-32**	**-18**	**-8**
Middle frontal gyrus	**R**	**13**	**0.030**	**4.97**	**46**	**46**	**20**
Thalamus proper	**L**	**2**	**0.031**	**4.96**	**-16**	**-34**	**2**
Lateral orbital gyrus	**L**	**3**	**0.033**	**4.94**	**-32**	**36**	**-16**
Hippocampus	**L**	**1**	**0.046**	**4.84**	**-32**	**-12**	**-12**

2.2 Supplementary Figures

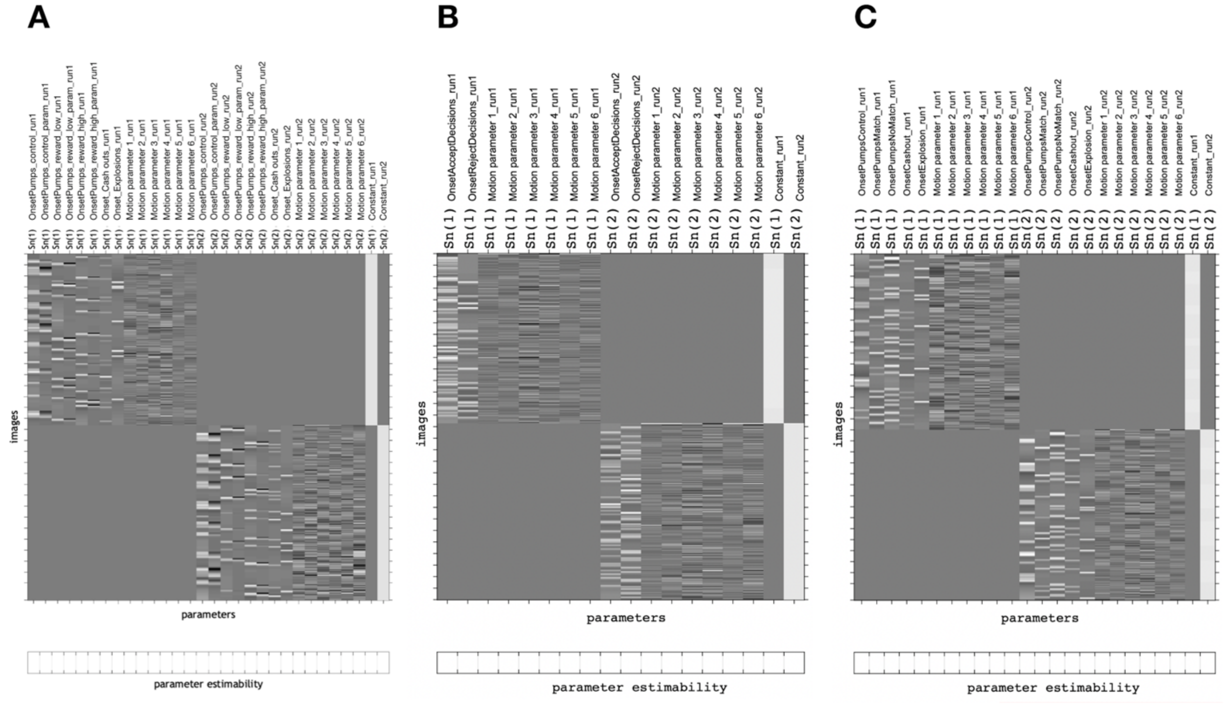

Supplementary Figure 1. Example individual-level design matrices to estimate neural activation differences. (A) Example design matrix for BART facilitating the main contrast analysis (‘Pumps reward versus Pumps control’). **(B)** Example design matrix for monetary gambles. **(C)** Example design matrix for BART facilitating supplementary average contrast analysis (‘Pumps reward versus Cash out’).

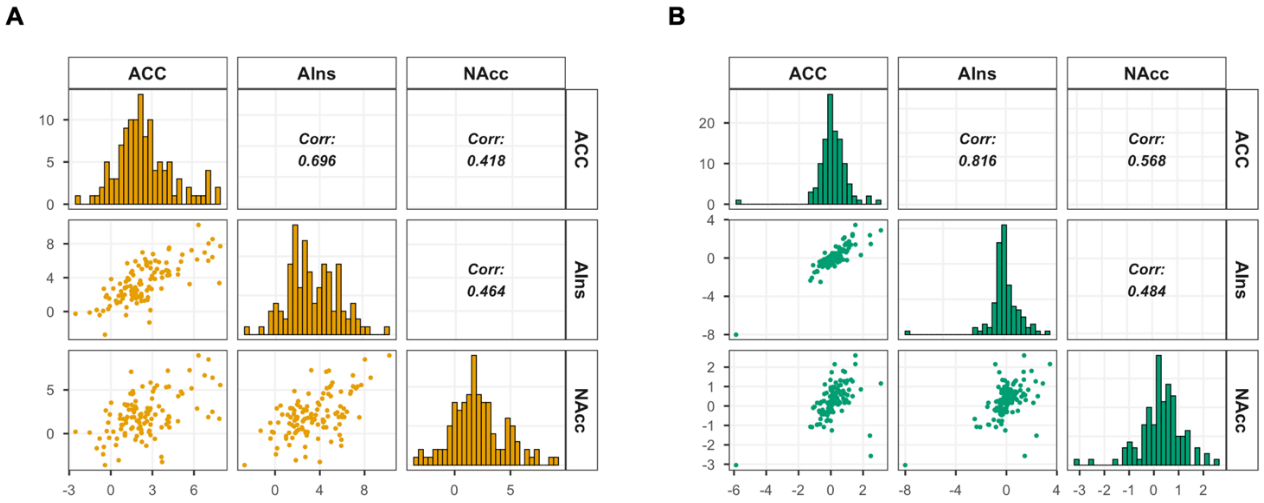

Supplementary Figure 2. Distribution of mean activation differences extracted from *risk matrix* ROIs for BART and monetary gambles. (A) Mean activation differences extracted from BART ‘Pumps reward > Pumps control’ contrast. **(B)** Mean activation differences extracted from monetary gambles ‘Accept > Reject’ contrast.

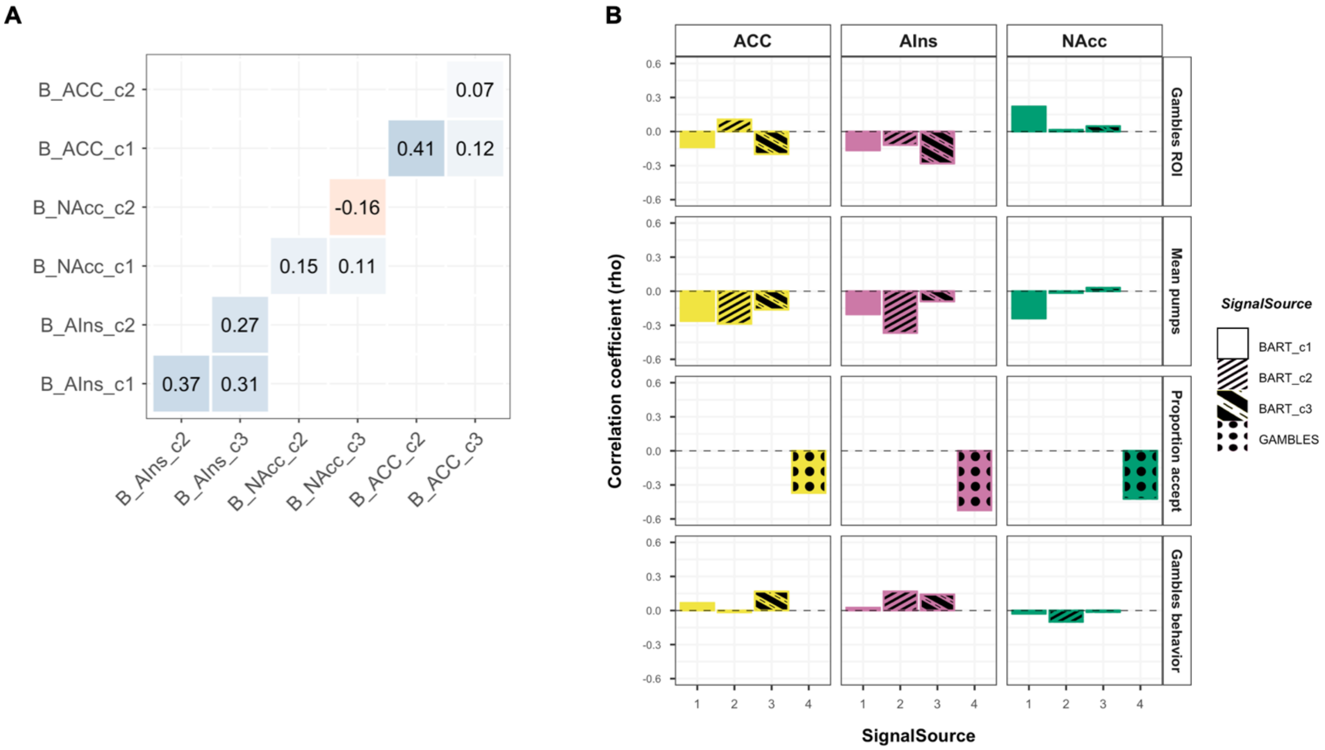

Supplementary Figure 3. Additional analytical approaches to within-session individual differences analyses for the BART. (A) Correlation between mean signal extracted from different BART contrasts. B = BART; ACC = anterior cingulate cortex, AIns = anterior insula, NAcc = nucleus accumbens; c1 = BART 'Pumps reward > Pumps control', c2 = BART 'Parametric pumps reward > Parametric pumps control', c3 = BART 'Pumps reward > Cash out'. **(B)** Visualization of the variability of within-session individual differences analyses (y-axis) as a function of BART contrast ('SignalSource' on the x-axis), labels as in panel A. Pendant to Figure 4 in main text, with regression coefficients from Figure 4 being shown in each plot for comparison (outer left bar, 'BART_c1'). For comparison and to mirror the layout of Figure 4 in the main text, plots in the third row show the original results for monetary gambles.

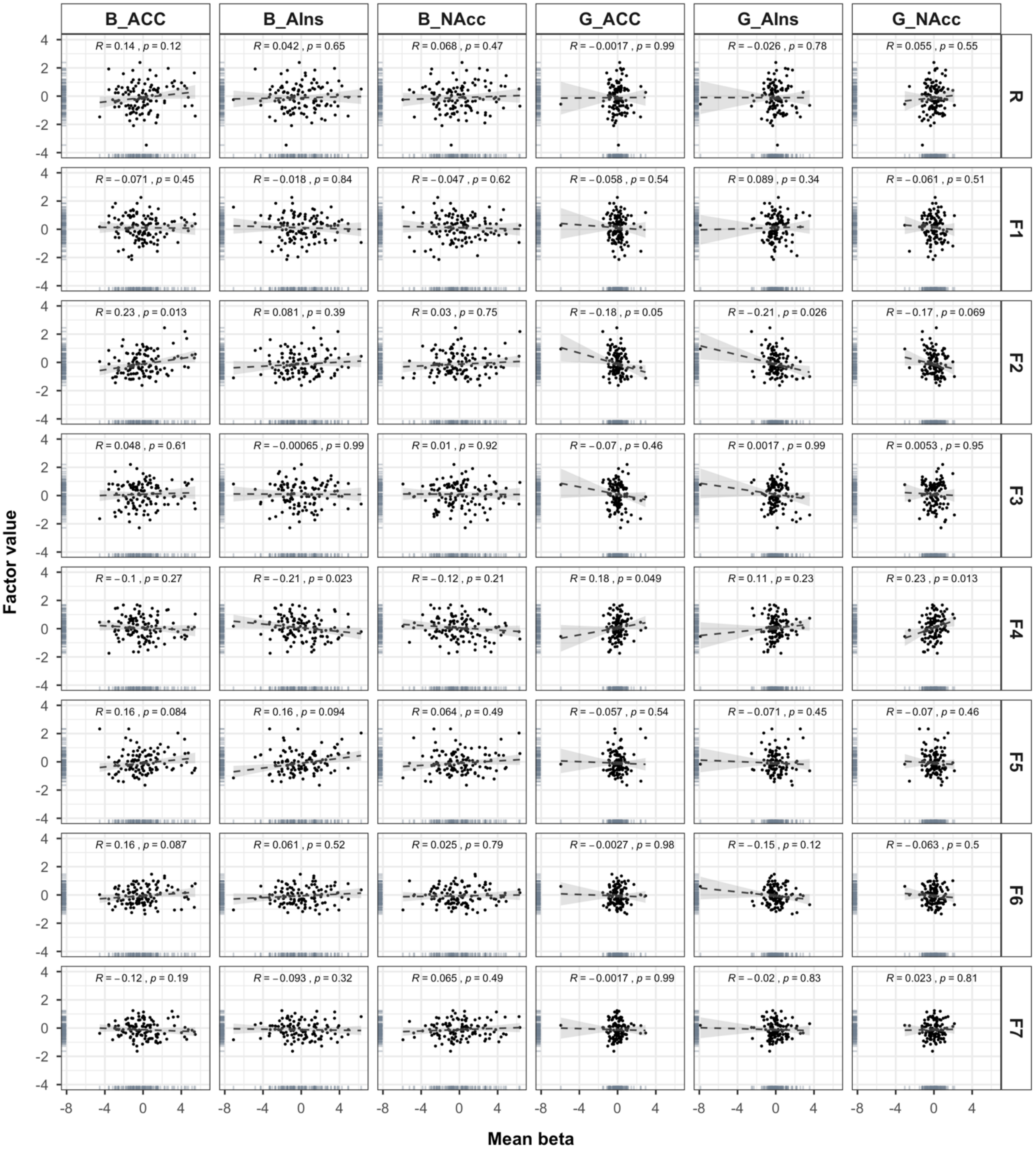

Supplementary Figure 4. Scatterplots for brain-behavior associations for risk preference factors. Plotted regression slopes were estimated using robust regression analysis. R = Spearman's rank-order correlation coefficient rho. B = BART ('Pumps reward > Pumps control'), G = monetary gambles ('Accept > Reject'); ACC = anterior cingulate cortex, AIns = anterior insula, NACC = nucleus accumbens; R = general risk-preference factor, F1 = health risk taking, F2 = financial risk taking, F3 = recreational risk taking, F4 = impulsivity, F5 = traffic risk taking, F6 = occupational risk

taking, F7 = choices among (monetary) lotteries. All analyses were performed on residualized variables (with respect to effects of age and gender for neural activation, and age, gender and study site for risk preference factors).

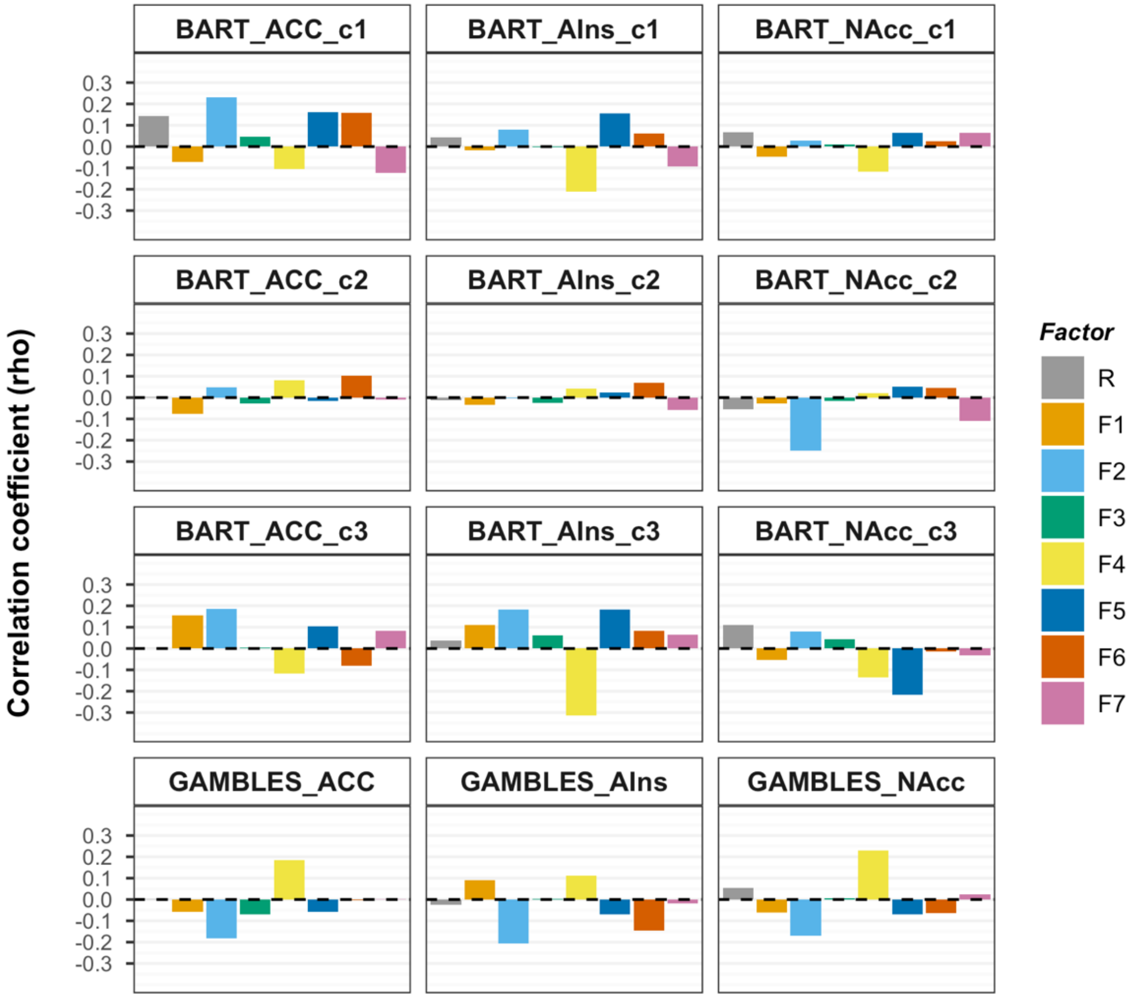

Supplementary Figure 5. Additional analytical approaches to brain-behavior associations involving risk preference factors. On the y-axis we plot the correlation coefficients (rho), from all out-of-session brain-behavior associations involving the psychometric risk preference factors, on the x-axis are the different risk preference factors (see legend). Panels are organized by fMRI contrast analyses. ACC = anterior cingulate cortex, AIns = anterior insula, NAcc = nucleus accumbens; c1 = BART ‘Pumps reward > Pumps control’, c2 = BART ‘Parametric pumps reward > Parametric pumps control’, c3 = BART ‘Pumps reward > Cash out’. Pendant to Figure 5C in the main text. R = general risk-preference factor, F1 = health risk taking, F2 = financial risk taking, F3 = recreational risk taking, F4 = impulsivity, F5 = traffic risk taking, F6 = occupational risk taking, F7 = choices among (monetary) lotteries.

References

Ashburner, John, and Karl J. Friston. 2005. "Unified Segmentation." *NeuroImage* 26 (3): 839–51. https://doi.org/10.1016/j.neuroimage.2005.02.018.

Barkley-Levenson, Emily E., Linda Van Leijenhorst, and Adriana Galván. 2013. "Behavioral and Neural Correlates of Loss Aversion and Risk Avoidance in Adolescents and Adults." *Developmental Cognitive Neuroscience* 3 (1): 72–83. https://doi.org/10.1016/j.dcn.2012.09.007.

Chang, Luke J., Tal Yarkoni, Mel Win Khaw, and Alan G. Sanfey. 2013. "Decoding the Role of the Insula in Human Cognition: Functional Parcellation and Large-Scale Reverse Inference." *Cerebral Cortex* 23 (3): 739–49. https://doi.org/10.1093/cercor/bhs065.

Helfinstein, Sarah M., Tom Schonberg, Eliza Congdon, Katherine H. Karlsgodt, Jeanette A. Mumford, Fred W. Sabb, Tyrone D. Cannon, Edythe D. London, Robert M. Bilder, and Russell A. Poldrack. 2014. "Predicting Risky Choices from Brain Activity Patterns." *Proceedings of the National Academy of Sciences of the United States of America* 111 (7): 2470–75. https://doi.org/10.1073/pnas.1321728111.

Knutson, Brian, and Scott A. Huettel. 2015. "The Risk Matrix." *Current Opinion in Behavioral Sciences* 5: 141–46. https://doi.org/10.1016/j.cobeha.2015.10.012.

Lejuez, C W, Jennifer P Read, Christopher W Kahler, Jerry B Richards, Susan E Ramsey, Gregory L Stuart, David R Strong, and Richard A Brown. 2002. "Evaluation of a Behavioral Measure of Risk Taking: The Balloon Analogue Risk Task (BART)." *Journal of Experimental Psychology. Applied* 8 (2): 75–84. https://doi.org/10.1037/1076-898X.8.2.75.

Namkung, Ho, Sun-hong Kim, and Akira Sawa. 2017. "The Insula: An Underestimated Brain Area in Clinical Neuroscience, Psychiatry, and Neurology." *Trends in Neurosciences* 40 (4): 200–207. https://doi.org/10.1016/j.tins.2017.02.002.

Rao, Hengyi, Marc Korczykowski, John Pluta, Angela Hoang, and John A. Detre. 2008. "Neural Correlates of Voluntary and Involuntary Risk Taking in the Human Brain: An FMRI Study of the Balloon Analog Risk Task (BART)." *NeuroImage* 42 (2): 902–10. https://doi.org/10.1016/j.neuroimage.2008.05.046.

Schonberg, Tom, Craig R. Fox, Jeanette A. Mumford, Eliza Congdon, Christopher Trepel, and Russell A. Poldrack. 2012. "Decreasing Ventromedial Prefrontal Cortex Activity during Sequential Risk-Taking: An FMRI Investigation of the Balloon Analog Risk Task." *Frontiers in Neuroscience* 6 (June): 1–11. https://doi.org/10.3389/fnins.2012.00080.

Schonberg, Tom, Craig R. Fox, and Russell A. Poldrack. 2011. "Mind the Gap: Bridging Economic and Naturalistic Risk-Taking with Cognitive Neuroscience." *Trends in Cognitive Sciences* 15 (1): 11–19. https://doi.org/10.1016/j.tics.2010.10.002.
